# Phase-Locked Inhibition, but Not Excitation, Underlies Hippocampal Ripple Oscillations in Awake Mice In Vivo

**DOI:** 10.1016/j.neuron.2016.12.018

**Published:** 2017-01-18

**Authors:** Jian Gan, Shih-ming Weng, Alejandro J. Pernía-Andrade, Jozsef Csicsvari, Peter Jonas

**Affiliations:** 1 IST Austria (Institute of Science and Technology Austria), Am Campus 1, A-3400 Klosterneuburg, Austria

**Keywords:** sharp wave-ripples, in vivo recording, in vivo voltage clamp, EPSCs, IPSCs, hippocampus, CA1 region, network oscillations, PV^+^ interneurons, GABAergic synapses

## Abstract

Sharp wave-ripple (SWR) oscillations play a key role in memory consolidation during non-rapid eye movement sleep, immobility, and consummatory behavior. However, whether temporally modulated synaptic excitation or inhibition underlies the ripples is controversial. To address this question, we performed simultaneous recordings of excitatory and inhibitory postsynaptic currents (EPSCs and IPSCs) and local field potentials (LFPs) in the CA1 region of awake mice in vivo. During SWRs, inhibition dominated over excitation, with a peak conductance ratio of 4.1 ± 0.5. Furthermore, the amplitude of SWR-associated IPSCs was positively correlated with SWR magnitude, whereas that of EPSCs was not. Finally, phase analysis indicated that IPSCs were phase-locked to individual ripple cycles, whereas EPSCs were uniformly distributed in phase space. Optogenetic inhibition indicated that PV^+^ interneurons provided a major contribution to SWR-associated IPSCs. Thus, phasic inhibition, but not excitation, shapes SWR oscillations in the hippocampal CA1 region in vivo.

## Highlights

• High-resolution synaptic current recording during sharp wave-ripples (SWRs) in vivo
• Inhibition dominates over excitation during SWRs in the hippocampal CA1 region
• Phasic inhibition, but not excitation, is phase-locked to individual ripple cycles
• PV^+^ interneurons substantially contribute to SWR-associated inhibitory conductance

## Teaser

Using high-resolution whole-cell recording of postsynaptic current from CA1 pyramidal neurons in awake mice, Gan et al. find that during sharp wave-ripples, inhibition dominates over excitation, is phase-locked to individual ripple cycles, and is primarily mediated by PV^+^ interneurons.

## Introduction

Sharp wave-ripple (SWR) oscillations in the hippocampal CA1 region play a key role in memory consolidation during non-rapid eye movement sleep, immobility, and consummatory behavior (Buzsáki et al., 1992, Ylinen et al., 1995, Girardeau et al., 2009, Jadhav et al., 2012; reviewed by Buzsáki, 2015). Despite their important network function, the underlying synaptic mechanisms remain unclear. Several mechanisms of ripple generation have been proposed, including phasic excitation from the CA3 region (Maier et al., 2011), phasic inhibition from GABAergic interneurons (English et al., 2014), and gap junction coupling between pyramidal neurons (Draguhn et al., 1998). Divergent results may derive from technical limitations of the experimental approaches used. Extracellular local field potential (LFP) recording in vivo (Ylinen et al., 1995, Stark et al., 2014) can be applied to networks in awake, behaving animals, but does not allow accurate dissection of dendritic excitation and perisomatic inhibition, which will produce similar sink-source patterns. Intracellular analysis allows precise analysis of synaptic potentials, currents, and spiking, but is largely restricted to in vitro preparations (Hájos et al., 2013, Schlingloff et al., 2014), or to the analysis of synaptic potentials in vivo with limited temporal resolution (English et al., 2014, Hulse et al., 2016). Understanding the synaptic mechanisms of SWR generation requires recording of synaptic currents in CA1 pyramidal neurons in vivo in awake animals under voltage-clamp conditions, where excitation and inhibition can be precisely dissected (Borg-Graham et al., 1998).

## Results

**Figure 1:**
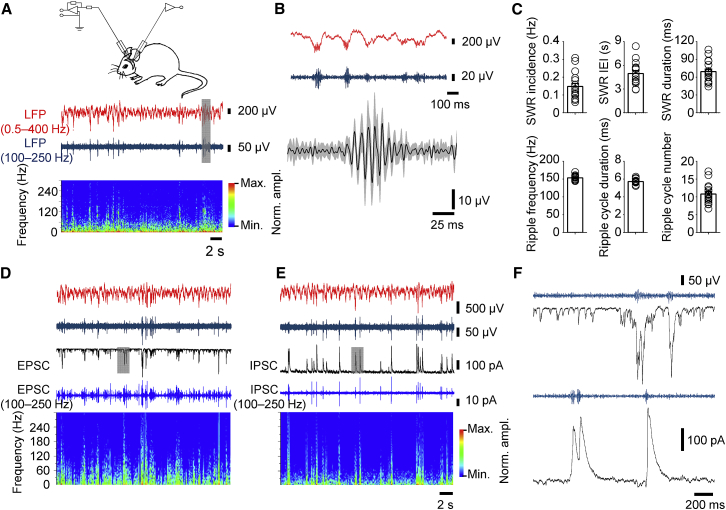
EPSCs and IPSCs Are Associated with SWRs in CA1 Pyramidal Neurons of Awake, Behaving Mice (A) Top, wide band (red; 0.5–400 Hz) and band pass-filtered LFP trace (blue; 100–250 Hz). Bottom, frequency-time plot of the LFP with amplitude indicated by the color code (log scale; calibration bar on the right). (B) Top, expanded view of wide band and band pass-filtered LFP trace (corresponding to the shaded area in A). Bottom, average of 26 SWRs (all SWRs recorded in one cell of a single awake mouse). Black line represents average trace; gray band indicates SEM. Individual SWRs were horizontally aligned to the maximal positive ripple deflection in the band pass-filtered LFP before averaging. (C) Summary bar graphs of SWR properties. Top, SWR incidence, inter-event interval (IEI), and SWR duration. Bottom, ripple frequency, ripple cycle duration, and ripple cycle number per sharp wave. Bars represent mean ± SEM; circles indicate data from individual LFP recordings. (D) Top, wide band (red; 0.5–400 Hz) and band pass-filtered LFP trace (blue; 100–250 Hz). Center, wide band EPSC trace (voltage clamp, black) and band pass-filtered EPSC trace (blue; 100–250 Hz). Holding potential was set to −70 mV to record EPSCs in isolation. Bottom, frequency-time plot of EPSC trace, with amplitude depicted by the color code (log scale; calibration bar in E, right). (E) Similar plots as shown in (D), but for IPSCs. Holding potential was set to +10 mV to isolate IPSCs. (F) Expanded view of EPSCs (top) and IPSCs (bottom) during SWRs (corresponding to the shaded areas in D and E). Data in (D)–(F) were recorded from the same cell. Note that in the example traces IPSCs were more tightly correlated to the SWRs than EPSCs.

To probe the synaptic mechanisms of SWR oscillations, we performed simultaneous recordings of excitatory and inhibitory synaptic currents (EPSCs and IPSCs) and LFPs in the CA1 region of awake mice in vivo (Figure 1). Animals were head-fixed, but fully awake, showing behaviors of grooming, whisking, and moving. We first tested whether functional characteristics of SWRs measured near the pyramidal cell layer under our experimental conditions were identical to those in awake, freely moving animals (Ylinen et al., 1995; Figures 1A–1C). On average, SWRs occurred with a mean frequency of 0.15 ± 0.02 Hz (694 events in 17 mice) and had a mean duration of 69.4 ± 4.3 ms. Individual ripple cycles were generated at a mean frequency of 154.0 ± 1.6 Hz, and the average number of ripples per sharp wave was 10.8 ± 0.7. These properties of SWRs were very similar to those reported previously in awake, behaving rodents (Ylinen et al., 1995; reviewed by Buzsáki, 2015).

Next, we investigated the postsynaptic currents during SWR oscillations in CA1 pyramidal neurons in the voltage-clamp configuration (Figures 1D–1F). To obtain adequate resolution, several efforts were made to optimize voltage-clamp conditions and to rigorously assess possible errors. First, we minimized series resistance (R_s_), which on average was 17 ± 1 MΩ in our dataset (range, 10–27 MΩ; Margrie et al., 2002, Lee et al., 2006, Lee et al., 2009). Additionally, we used a cesium-based internal solution to enhance steady-state voltage control in distal dendrites (Williams and Mitchell, 2008). Second, we performed experimental tests for adequate voltage-clamp conditions. Measured spontaneous synaptic events showed a fast time course (Figure S1, available online), and extrapolation toward the limit of small R_s_ revealed even faster synaptic kinetics (Figure S2). Kinetic parameters and amplitude were only weakly correlated. Both results were consistent with adequate clamp conditions (Hestrin et al., 1990; Figure S2). Finally, we examined the extent of voltage-clamp errors for EPSCs and IPSCs in detailed cable models based on full reconstruction of in vivo-recorded CA1 pyramidal neurons (Major et al., 1994; Figures S3–S5). For proximal synapses, errors in peak amplitude, rise time, and decay time constant of somatically recorded EPSCs and IPSCs due to cable filtering were less than a factor of two (Figure S3), and corresponding postsynaptic voltage errors were <10 mV (Figure S4). Distributed pipette capacitance did not introduce additional errors (Figure S5). In conclusion, voltage-clamp errors were unavoidable, but appeared to be within an acceptable range under our experimental conditions.

Finally, we dissected EPSCs and IPSCs during SWRs by alternatingly setting the holding potential to either −70 mV (i.e., near the reversal potential of GABA_A_ receptors; Figure S6) or +10 mV (i.e., near the reversal potential of AMPA- and NMDA-type glutamate receptors; Borg-Graham et al., 1998). Simultaneous recording of either EPSCs or IPSCs and the LFP revealed that the frequency of both excitatory and inhibitory events increased during SWRs (Figures 1D–1F). However, the generation of IPSCs (Figure 1E) appeared to be more tightly correlated to the SWRs than the generation of EPSCs (Figure 1D).

**Figure 2:**
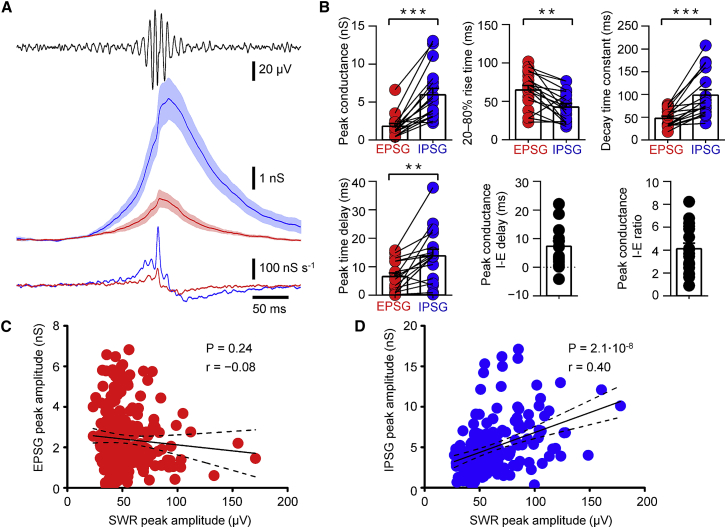
Inhibition Dominates over Excitation during SWRs and Correlates with Their Peak Amplitude (A) SWR-triggered average of excitatory (red) and inhibitory (blue) conductance. Top, average band pass-filtered LFP trace (100–250 Hz); center, mean excitatory and inhibitory conductance; bottom, corresponding first derivatives. Red and blue lines indicate mean from 17 cells; light red and light blue areas represent SEM values. Excitatory and inhibitory conductance (EPSG and IPSG) was calculated from average EPSCs and IPSCs by division of currents by driving force. Note that the first derivative of IPSGs shows high-frequency oscillations. (B) Summary bar graphs of SWR-triggered conductance properties. Top, peak conductance, 20%–80% rise time, and decay time constant of synaptic conductance. Bottom, delay between SWR center and peak of excitatory and inhibitory conductance, delay between peak of inhibitory and excitatory conductance, and corresponding conductance ratio. Bars represent mean ± SEM; circles indicate data from individual cells (red, excitatory events; blue, inhibitory events; black, data applying to both). Data from the same cell are connected by lines. (C and D) Scatterplots of peak SWR-associated excitatory (C) and inhibitory postsynaptic peak conductance (D) against SWR peak amplitude. Each point represents an individual SWR. Continuous line represents the results of linear regression to the data points; dashed lines indicate 95% confidence intervals. There are a total of 238 SWRs in (C) and 185 SWRs in (D). Data are from ten cells in which SWR amplitude varied over a wide range. Note the lack of correlation between EPSG peak amplitude and SWR peak amplitude (C) and the highly significant positive correlation between IPSG peak amplitude and SWR peak amplitude (D).

To quantitatively analyze the contribution of excitation and inhibition to the generation of ripples, we performed SWR-triggered averaging of EPSCs and IPSCs (Figure 2). Synaptic currents were aligned to the maximal positive ripple deflection in the band pass-filtered LFP, averaged, and converted into synaptic conductances using the values of the driving force. SWR-triggered averaging revealed that the peak amplitude of the inhibitory conductance was markedly larger than that of the excitatory conductance (Figure 2A). On average, the SWR-associated peak conductance was 1.8 ± 0.4 nS for excitation and 6.0 ± 0.8 nS for inhibition (17 cells; p < 0.0001; Figures 2A and 2B), corresponding to an inhibition-to-excitation conductance ratio of 4.1 ± 0.5. In comparison to excitation, inhibition showed a faster rise (20%–80% rise time, 64.7 ± 5.9 ms for excitation and 42.5 ± 4.8 ms for inhibition; p = 0.0021; Figures 2A and 2B), but a slower decay (decay time constant, 47.6 ± 4.6 ms for excitation and 98.3 ± 12.1 ms for inhibition; p < 0.0001; Figures 2A and 2B). Furthermore, inhibition peaked at a slightly later time (time difference, 7.3 ± 1.9 ms; p = 0.0005). Thus, synaptic inhibition, rather than excitation (Maier et al., 2011), dominated during the generation of SWRs. Furthermore, excitation and inhibition showed different temporal profiles.

Previous studies revealed that the amplitude of SWRs varies from event to event by over an order of magnitude (Csicsvari et al., 2000). If either excitation or inhibition was responsible for ripple generation, we would expect that the relevant conductance would positively correlate with the ripple amplitude. To test this hypothesis, we plotted the peak amplitude of excitatory and inhibitory conductance in CA1 pyramidal neurons during individual SWRs against ripple amplitude (Figures 2C and 2D). For the excitatory conductance, peak conductance and ripple amplitude were not significantly correlated (linear correlation analysis, Pearson’s r = −0.08; 238 events; p = 0.24; Figure 2C). In contrast, for the inhibitory conductance, peak conductance and ripple amplitude exhibited a strong positive correlation (Pearson’s r = 0.40; 185 events; p < 0.0001; Figure 2D). Thus, the magnitude of inhibition, but not that of excitation, was correlated with the amplitude of individual SWRs. Furthermore, excitation and inhibition were not strictly balanced during SWRs (Hulse et al., 2016).

**Figure 3:**
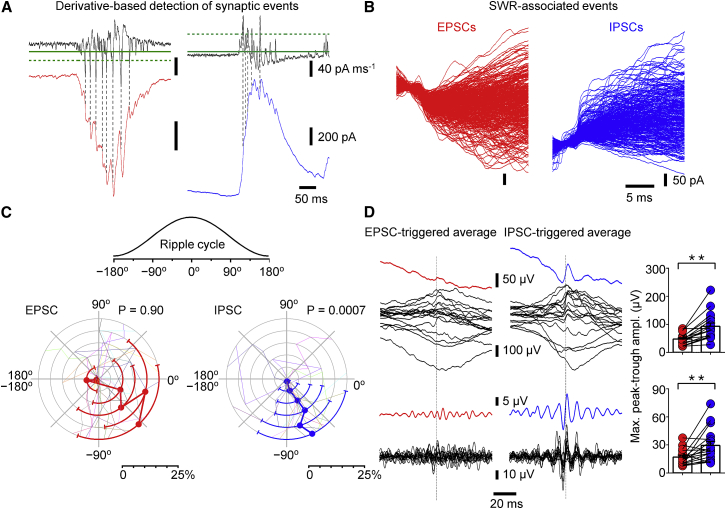
IPSCs, but Not EPSCs, Are Phase-Coupled to Ripple Oscillations (A) Representative EPSCs at −70 mV (left) and IPSCs at +10 mV (right) associated with SWRs. Upper trace indicates first derivative of EPSC/IPSC traces; green horizontal lines represent 25% (continuous) and 10% (dashed) highest minima (left) or maxima (right) in the derivative trace. Gray vertical dashed lines indicate events detected using the maximal of the derivative trace. For details, see Experimental Procedures. (B) Overlay of 372 detected EPSCs (left) and 278 detected IPSCs (right) during SWRs in a representative cell. (C) Polar plot of mean phase of EPSC (left) and IPSC onset (right), as revealed by peak derivative detection. Concentric rings indicate results for different percentages of largest derivative peaks (1%, 5%, 10%, 15%, 20%, and 25%). Colored thin lines represent data from individual cells, red and blue symbols and lines indicate mean, and error bars plotted on top of concentric rings represent angular deviation. Left, analysis for EPSCs (red); right, similar data for IPSCs (blue). Data are from 17 cells. Inset on top illustrates phase angle conventions. Note that the angular deviations are much smaller for IPSCs than for EPSCs, consistent with differential phase locking. p values indicate results from Rayleigh test for 10% largest derivative peaks. (D) EPSC/IPSC-triggered averaging of LFP traces. Left, analysis for EPSCs; center, similar analysis for IPSCs, for 10% largest derivative peaks in both cases. Top, wide band average LFPs (0.5–400 Hz). Bottom, band pass-filtered average LFPs (100–250 Hz). Black thin lines represent data from individual cells; colored lines indicate average (red, EPSC-triggered average; blue, IPSC-triggered average). Dashed vertical lines indicate EPSC and IPSC onset points, respectively. Right, summary bar graphs of maximal peak-to-trough amplitude of EPSC/IPSC-triggered average (red, EPSC; blue, IPSC; upper panels, wide band average LFPs [0.5–400 Hz]; bottom panels, band pass-filtered average LFPs [100–250 Hz]). Note that the band pass-filtered signal showed marked periodicity for IPSCs, but not for EPSCs.

To determine whether EPSCs and IPSCs were phase-locked to individual ripple cycles, we performed interleaved phase analysis for both EPSCs and IPSCs in CA1 pyramidal neurons (Figure 3). EPSC onset times were detected as minima of the first derivative of the membrane current at –70 mV, whereas IPSC onset times were determined as maxima of the first derivative at +10 mV (see Experimental Procedures; 3,566 and 3,180 events total for 25% largest derivative extrema; 17 cells; Figures 3A and 3B). Simulations using cable models based on cell reconstructions suggested that adequate event detection was achieved under these conditions (Figure S7). After computing the Hilbert transform of the band pass-filtered simultaneously recorded LFP signal (100–250 Hz; English et al., 2014), the onset times of EPSCs and IPSCs were individually assigned corresponding phase values, with 0° corresponding to the peak of a ripple. Whereas EPSCs were uniformly distributed (p = 0.90 for 10% largest derivative extrema; Rayleigh test; Figure 3C, left), IPSCs were significantly clustered in the late ascending phase of ripple cycles (p = 0.0007 for 10% largest derivative extrema; Rayleigh test; mean phase angle, −62.9° ± 49.6°, equivalent to 297.1°; Figure 3C, right). This conclusion was corroborated by reverse analysis using EPSC- or IPSC-triggered averaging of the LFP (Figure 3D). Synaptic event-triggered LFP averages were weakly rhythmically modulated for EPSC onsets (Figure 3D, left), but strongly modulated for IPSC onsets (Figure 3D, right). The peak-to-trough amplitude of the triggered LFP average was significantly larger for IPSCs than for EPSCs (p < 0.0001 and 0.0038). Thus, phasic inhibition, but not excitation, was phase-locked to individual cycles of ripple oscillations.

**Figure 4:**
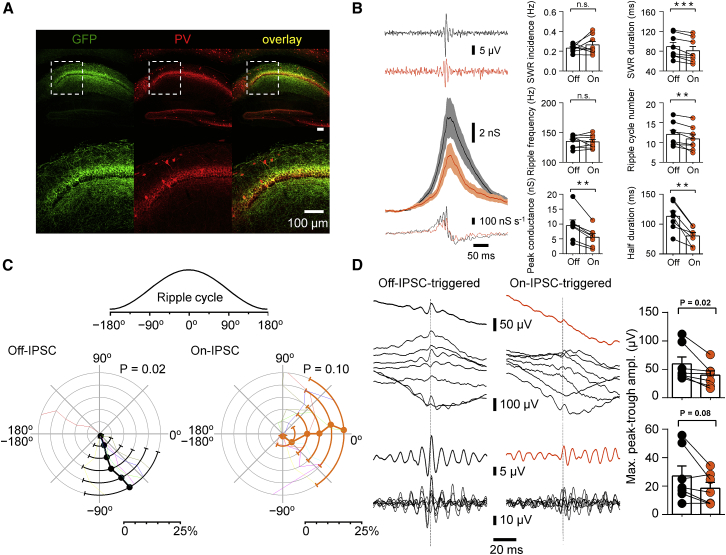
Optogenetic Suppression of PV^+^ Interneurons Reduces SWR-Associated Inhibitory Conductance and Disrupts Phase Preference of IPSCs (A) AAV infection leads to selective expression of ArchT-GFP in PV^+^ interneurons of the CA1 region. Maximum intensity projection of confocal stacks. Left, GFP fluorescence; center, immunoreactivity for PV; right, overlay. Note the high degree of co-localization. (B) Left, SWR-triggered average of inhibitory conductance in control period (black) and during laser illumination (orange). Top, average band pass-filtered LFP trace (100–250 Hz); center, mean inhibitory conductance from seven cells; bottom, corresponding first derivative. Gray and light orange areas represent SEM values. Inhibitory conductance was calculated from average IPSCs dividing current by driving force. Note that the first derivative of IPSGs in the absence of light shows high-frequency oscillations. Right, summary bar graphs of SWR properties (top four graphs) and inhibitory conductance properties (bottom two graphs). Black, laser illumination off; orange, laser illumination on. Note that optogenetic inhibition of PV^+^ interneurons significantly changes the properties of SWRs in the LFP and substantially reduces amplitude and duration of the inhibitory conductance. (C) Polar plot of mean phase of IPSC onset, as revealed by peak derivative detection. Concentric rings indicate results for different percentages of largest derivative peaks (1%, 5%, 10%, 15%, 20%, and 25%). Colored thin lines represent data from individual cells, black and orange symbols and lines indicate mean, and error bars plotted on top of concentric rings represent angular deviation. Left, analysis for epochs with laser illumination off (black); right, similar data for epochs with laser illumination on (orange). Data are from seven cells. Inset on top illustrates phase angle conventions. Note significant phase locking in the off periods (p = 0.02; left; similar to Figure 3C, right panel), but not in the on periods (p = 0.10; right). p values indicate results from Rayleigh test for 10% largest derivative peaks. (D) IPSC-triggered averaging of LFP traces. Left, analysis for epochs with laser illumination off (black); right, similar data for epochs with laser illumination on (orange) for 10% largest derivative peaks in both cases. Top, wide band average LFPs (0.5–400 Hz). Bottom, band pass-filtered average LFPs (100–250 Hz). Black thin lines represent data from individual cells; thick lines indicate averages (black, off epochs; orange, on epochs). Dashed vertical lines indicate IPSC onset points. Right, summary bar graphs of maximal peak-to-trough amplitude of IPSC-triggered average (black, off epochs; orange, on epochs; upper panels, wide band average LFPs [0.5–400 Hz]; bottom panels, band pass-filtered average LFPs [100–250 Hz]). Note that the IPSC-triggered LFP signals showed marked periodicity without laser illumination, but not with laser illumination.

As PV^+^ interneurons strongly fire during SWRs (Lapray et al., 2012, Varga et al., 2012; see Hájos et al., 2013, Schlingloff et al., 2014), they are possible candidates for the generation of the SWR-associated inhibitory conductance. To test this hypothesis, we examined the effects of optogenetic inhibition of PV^+^ interneurons (Figure 4). The inhibitory opsin Archaerhodopsin T (ArchT) was selectively expressed in PV^+^ interneurons in the CA1 region of the hippocampus (Figure 4A). Five-second laser pulses were alternated with 11 s off periods, and properties of SWRs and associated inhibitory postsynaptic conductance were compared between on and off periods (Figure 4B). Optogenetic inhibition of PV^+^ interneurons did not change the incidence of SWRs, but significantly reduced SWR duration (from 89.3 ± 8.5 ms to 81.0 ± 8.1 ms; 8 mice; p = 0.008; Figure 4B, right top). Furthermore, light pulses did not change the ripple frequency, but decreased the number of ripple cycles per SWR (from 12.1 ± 1.0 to 10.9 ± 1.0; p = 0.008; Figure 4B, right center). Finally, optogenetic inhibition of PV^+^ interneurons markedly reduced the peak amplitude of the SWR-associated inhibitory conductance (from 9.5 ± 1.9 nS to 5.6 ± 1.2 nS; 7 cells; p = 0.016) and reduced its half-duration (Figure 4B, right bottom). In conclusion, PV^+^ interneurons make a major contribution to the inhibitory conductance during SWRs. Furthermore, they control the duration of SWRs and the number of ripples in SWR complexes.

If PV^+^ interneurons are primarily responsible for SWR generation, optogenetic inhibition of these interneurons might result in a disruption of phase locking of IPSCs. To test this hypothesis, we performed phase analysis and IPSC-triggered LFP averaging before and after optogenetic inhibition after adeno-associated virus-mediated expression of ArchT (Figures 4C and 4D). In the absence of light, IPSCs were phase-locked to the ascending phase of the ripple cycle (Figure 4C, left), similar to the results in uninfected control animals (Figure 3C, right). In contrast, in the presence of light, the phase preference was perturbed (Figure 4C, right). Furthermore, the peak-to-trough amplitude of IPSC-triggered LFP averages was markedly reduced (Figure 4D). Thus, PV^+^ interneurons provide a major contribution to phase-locked IPSCs during individual ripple cycles.

## Discussion

The present paper provides a quantitative analysis of postsynaptic conductances during SWRs in hippocampal CA1 pyramidal neurons in awake, behaving mice in vivo. Our results indicate that during SWRs, (1) inhibition dominates over excitation; (2) phasic inhibition, but not excitation, is positively correlated with SWR amplitude; (3) phasic inhibition, but not excitation, is phase-locked to individual ripple cycles; and (4) PV^+^ interneurons provide a major contribution to the SWR-associated inhibitory conductance. Our findings directly reveal the current generator underlying SWRs. This was possible because our experiments provided information about both phase and location of the underlying conductance. The onset of the inhibitory conductance occurred at a phase of ∼−60°, corresponding to the ascending phase of a ripple. Furthermore, the conductance was likely to be perisomatic because it was mediated, to a large extent, by PV^+^ interneurons (Hu et al., 2014). Thus, the inhibitory conductance represents a perisomatic current source, resulting in a positive deflection in the LFP near the pyramidal cell layer.

Furthermore, our results shed light on the function of the rhythm generator. They suggest a model in which SWRs are generated by a combination of tonic excitation from CA3 and phasic inhibition within CA1. In contrast, our results are inconsistent with models in which phasic excitation is relayed from CA3 (Maier et al., 2011; see Nakashiba et al., 2009, Middleton and McHugh, 2016). Furthermore, they are incompatible with a major contribution of gap junctions (Draguhn et al., 1998). Whether ripple generation involves feedback inhibition, mutual inhibition, or both remains to be determined (Stark et al., 2014). The temporal sequence of events, with action potentials in CA1 pyramidal neurons at the ripple trough (Csicsvari et al., 1999), PV^+^ interneuron firing in the early ascending phase (Lapray et al., 2012, Varga et al., 2012), and onset of inhibitory conductance in the late ascending phase of the ripples (this paper), would be consistent with a feedback mechanism (Stark et al., 2014). On the other hand, one might expect that GABAergic interneurons receive tonic excitation during SWRs, similar to that in CA1 pyramidal neurons. This could activate mutual inhibition circuits, independent of feedback innervation.

Finally, our findings have implications for temporal coding of information in the hippocampal CA1 region. Importantly, they explain why CA1 pyramidal neurons in vivo fire at the ripple trough (Csicsvari et al., 1999). In a ripple model based on phasic inhibition, the descending phase of the ripple coincides with the decay of the inhibitory conductance. Pyramidal cells will fire at a point where inhibition has become minimal, which is near the trough in the LFP. In this scenario, fast decay of the inhibitory conductance in CA1 pyramidal neurons in vivo (Figure S1) will ensure temporally precise action potential generation in these neurons (Carter and Regehr, 2002). Precise spike timing, in turn, may be critically important for the preplay or replay of temporal activity sequences in cell assemblies (Skaggs and McNaughton, 1996) and for the consolidation of memory via spike timing-dependent plasticity in downstream neocortical target cells.

## Experimental Procedures

In vivo whole-cell patch-clamp recordings from CA1 pyramidal neurons and simultaneous LFP recordings were performed in head-fixed, fully awake mice. All experiments were carried out in strict accordance with institutional, national, and European guidelines for animal experimentation. Protocols were approved by the Bundesministerium für Wissenschaft und Forschung of Austria (BMWF-66.018/0008-II/3b/2010, BMWFW-66.018/0007-WF/II/3b/2014). PV^+^ interneurons were optogenetically manipulated using adeno-associated virus. EPSCs and IPSCs were detected using template- or derivative-based detection methods. Simulations were performed on detailed cable models of biocytin-filled CA1 pyramidal neurons. For details, see Supplemental Information.

## Author Contributions

J.G. planned experiments, performed experiments, and analyzed data, S.-m.W. performed initial experiments, A.J.P.-A. supervised initial experiments, J.C. and P.J. conceived the project, and P.J. performed simulations and wrote the paper. All authors jointly revised the paper.

## Supplemental Information

Document S1. Supplemental Experimental Procedures and Figures S1–S7

Document S2. Article plus Supplemental Information
