# Supplementary material for: Phase-Locked Inhibition, but Not Excitation, Underlies Hippocampal Ripple Oscillations in Awake Mice In Vivo
Source: Neuron. 2017 Jan 18;93(2):308–14. doi: 10.1016/j.neuron.2016.12.018 (PMC5263253; doi:10.1016/j.neuron.2016.12.018)
Supplement: Document S1. Supplemental Experimental Procedures and Figures S1–S7 [file mmc1.pdf]

Neuron, Volume *93*

Supplemental Information

Phase-Locked Inhibition, but Not Excitation, Underlies Hippocampal Ripple Oscillations in Awake Mice In Vivo

Jian Gan, Shih-ming Weng, Alejandro J. Pernía-Andrade, Jozsef Csicsvari, and Peter Jonas

Figure S1. Properties of spontaneous EPSCs and IPSCs in CA1 pyramidal neurons *in vivo*, related to Figure 1.

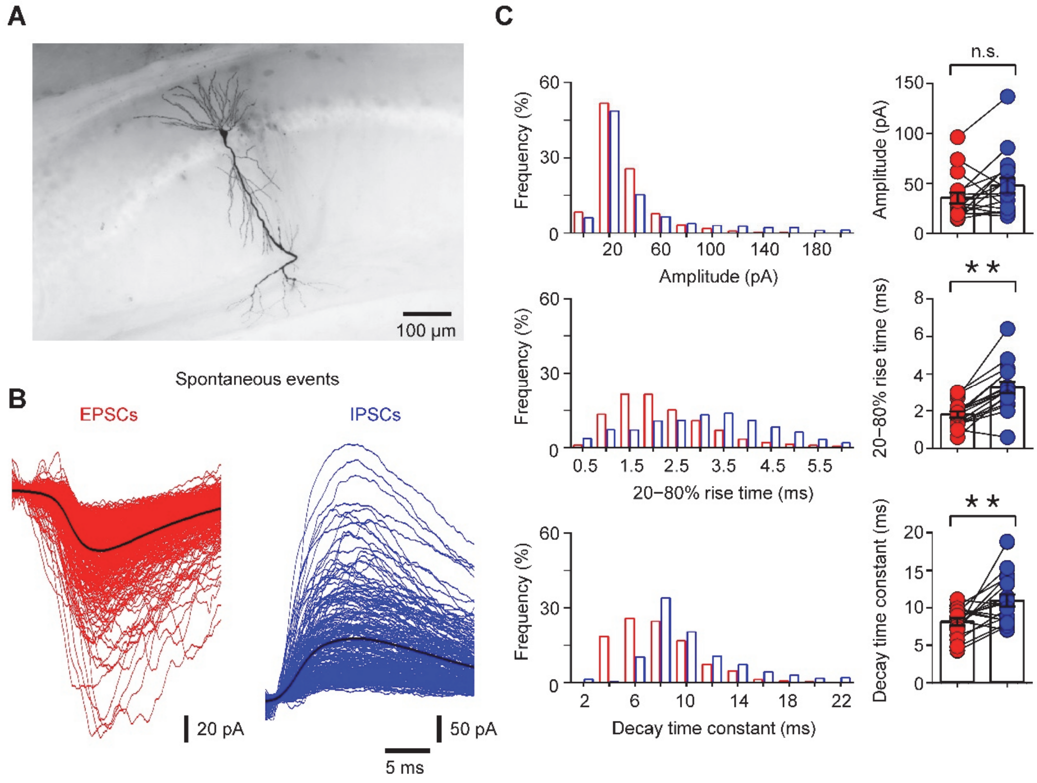

(A) Light micrograph of CA1 pyramidal neuron filled with biocytin during recording and visualized by 3,3'-diaminobenzidine.

(B) Superposition of 353 spontaneous EPSCs (left) and 322 spontaneous IPSCs (right). Data in A and B were obtained from the same cell. Holding potential was set to −70 mV and +10 mV to record EPSCs and IPSCs, respectively, in isolation.

(C) Peak amplitude, 20–80% rise time, and decay time constant of spontaneous EPSCs (red) and IPSCs (blue). Left, frequency distribution bar graphs of data from all cells (bin width: 20 pA, 0.5 ms, and 2 ms, respectively); right, summary bar graph obtained from individual experiments. Data from 17 cells.

Figure S2. Evidence for adequate voltage-clamp conditions in CA1 pyramidal neurons recorded *in vivo*, related to Figure 1.

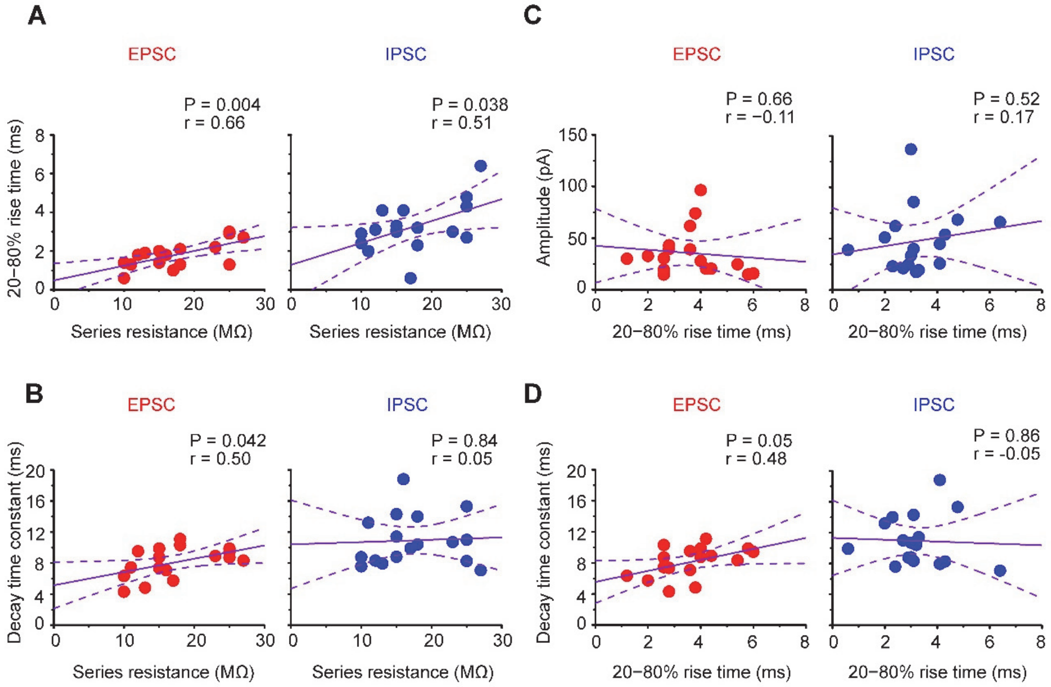

(A) Plot of 20–80% rise time against series resistance.

(B) Plot of decay time constant against series resistance. Extrapolation towards a series resistance of 0 may give information about the “true” kinetics of the synaptic currents.

(C) Scatter plot of peak amplitude versus 20–80% rise time. Left, EPSC data; right, IPSC data.

(D) Similar scatter plot of decay time constant versus rise time. Note lack of correlation or weak correlation (D, left), consistent with adequate voltage-clamp conditions (Hestrin et al., 1990).

Continuous lines represent the results of linear regression, dashed lines indicate the 95%-confidence intervals. Numbers indicate significance level P and corresponding Pearson’s r. Data from 17 cells. In addition to analysis of population data, scatter plots were also analyzed for individual EPSCs and IPSCs on a cell-by-cell basis. Significant negative correlations between peak amplitude and 20–80% rise time were obtained in only 4 out of 17 cells. Furthermore, significant positive correlations between decay time constant and 20–80% rise time were obtained in only 2 out of 17 cells.

Figure S3. Simulation of space clamp errors in cable model based on reconstruction of *in vivo* labeled CA1 pyramidal neuron, related to Figures 1, 2, 3, and 4.

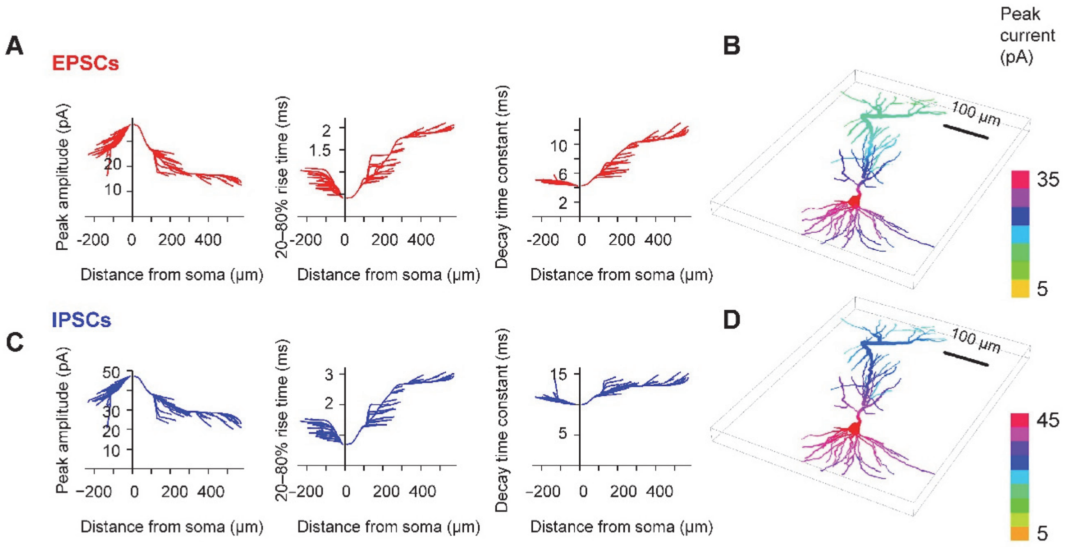

(A and B) Simulation of EPSCs (holding potential −70 mV, synaptic reversal potential 0 mV). Synapses were simulated at all possible dendritic locations, and somatic EPSCs were analyzed. (A) Plot of peak amplitude, 20–80% rise time, and decay time constant against distance (positive distance, apical dendrite; negative distance, basal dendrite). (B) Color-coded representation of the morphological properties of the CA1 pyramidal neuron, with color representing peak current amplitude of the somatic EPSC.

(C and D) Similar analysis as in (A) and (B), but for IPSCs (holding potential +10 mV, synaptic reversal potential −65 mV).

Synaptic peak conductance 1 nS; rise time constant 0.2 ms; decay time constant 2.5 ms (EPSCs) or 7.5 ms (IPSCs).

Figure S4. Simulation of postsynaptic voltage error during voltage-clamp experiments, related to Figures 1, 2, 3, and 4.

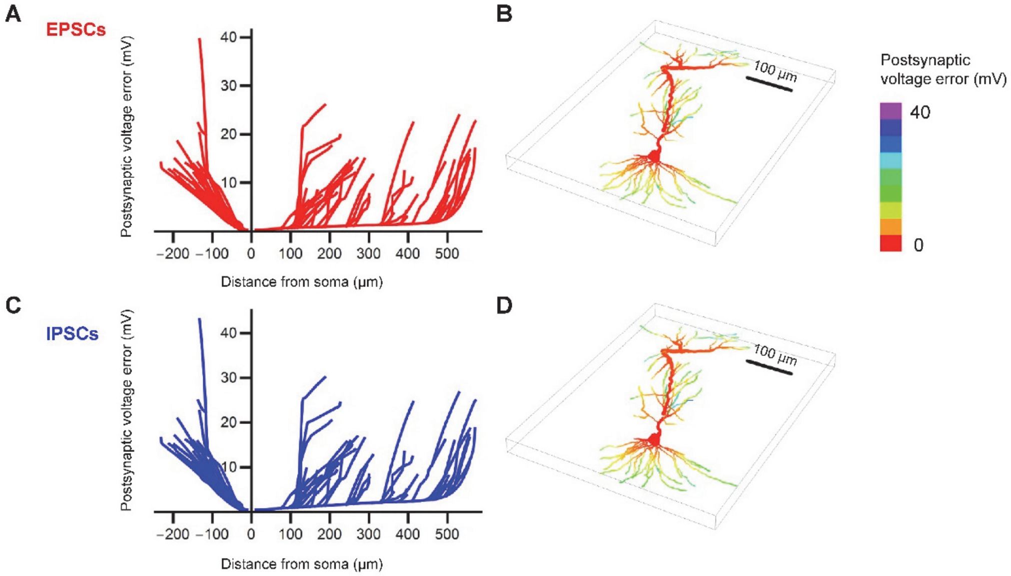

(A and B) Simulation of EPSCs (holding potential −70 mV, synaptic reversal potential 0 mV). Synapses were simulated at all possible dendritic locations, and somatic EPSCs were analyzed. (A) Plot of postsynaptic voltage change at the site of the synapse against distance (positive distance, apical dendrite; negative distance, basal dendrite). (B) Color-coded representation of the morphological properties of the CA1 pyramidal neuron, with color representing postsynaptic voltage error. Note that postsynaptic voltage error is small at proximal dendritic locations but increases near sealed ends.

(C and D) Similar analysis as in (A) and (B), but for IPSCs (holding potential +10 mV, synaptic reversal potential −65 mV).

Synaptic peak conductance 1 nS; rise time constant 0.2 ms; decay time constant 2.5 ms (EPSCs) or 7.5 ms (IPSCs).

Figure S5. Simulation of voltage-clamp errors with realistic pipette models, related to Figures 1, 2, 3, and 4.

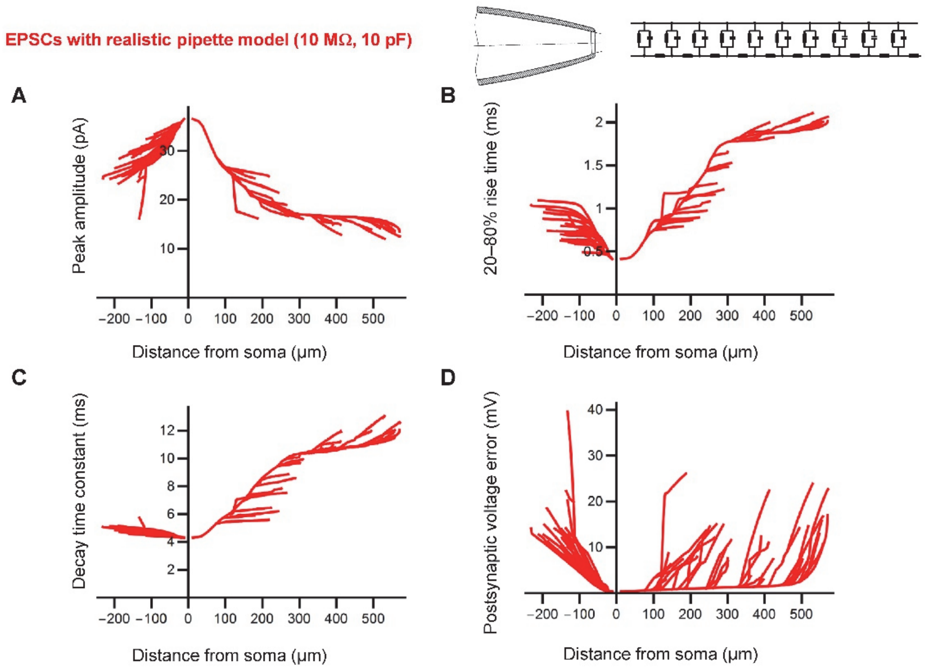

Simulation of EPSCs (holding potential −70 mV, synaptic reversal potential 0 mV). Synapses were simulated at all possible dendritic locations, and somatic EPSCs were analyzed. Plot of peak amplitude (A), 20–80% rise time (B), decay time constant (C), and postsynaptic voltage error (D) against distance (positive distance, apical dendrite; negative distance, basal dendrite). Pipette was implemented realistically as a multi-compartment structure; C_{pip} = 10 pF, R_s = 10 MΩ (scheme on top; 10 x 10 compartments). Results were almost identical to those without realistic pipette implementation (**Figure S3**A), indicating that errors introduced by the distributed nature of pipette capacitance and resistance were negligible.

Figure S6. Reversal potential of IPSCs in CA1 pyramidal neurons *in vivo*, evoked by optogenetic activation of PV^+ interneurons, related to Figures 1, 2, 3, and 4.

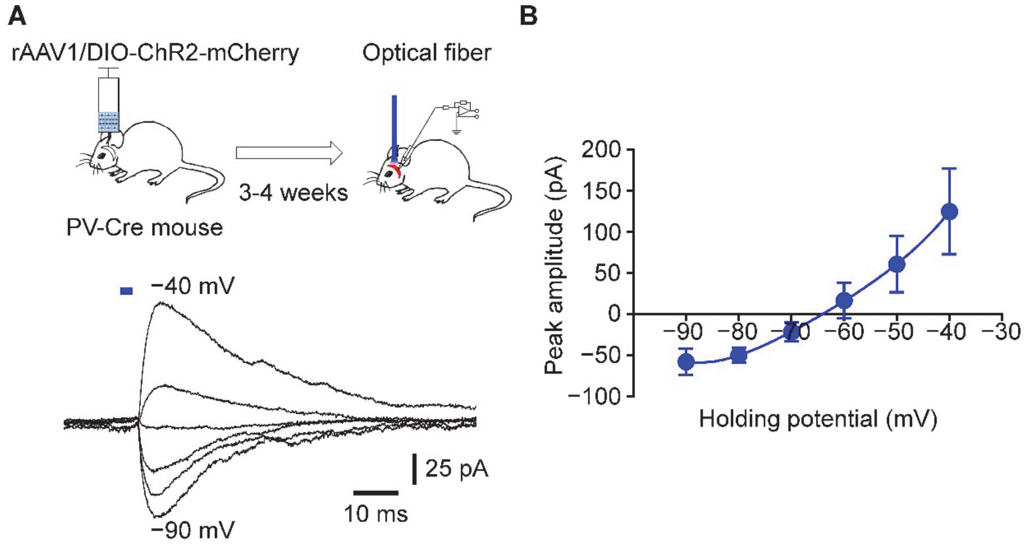

(A) Evoked IPSCs in CA1 pyramidal neurons at different holding potentials. Inset schematically shows whole-cell recording combined with optogenetic stimulation of PV^+ interneurons.

(B) Current–voltage relation of IPSCs. Data points were fit with 4th-order polynomial function. Data from 4 neurons (in different mice).

Figure S7. Simulation of accuracy of derivative-based method for detection of EPSCs and IPSCs, related to Figures 3 and 4.

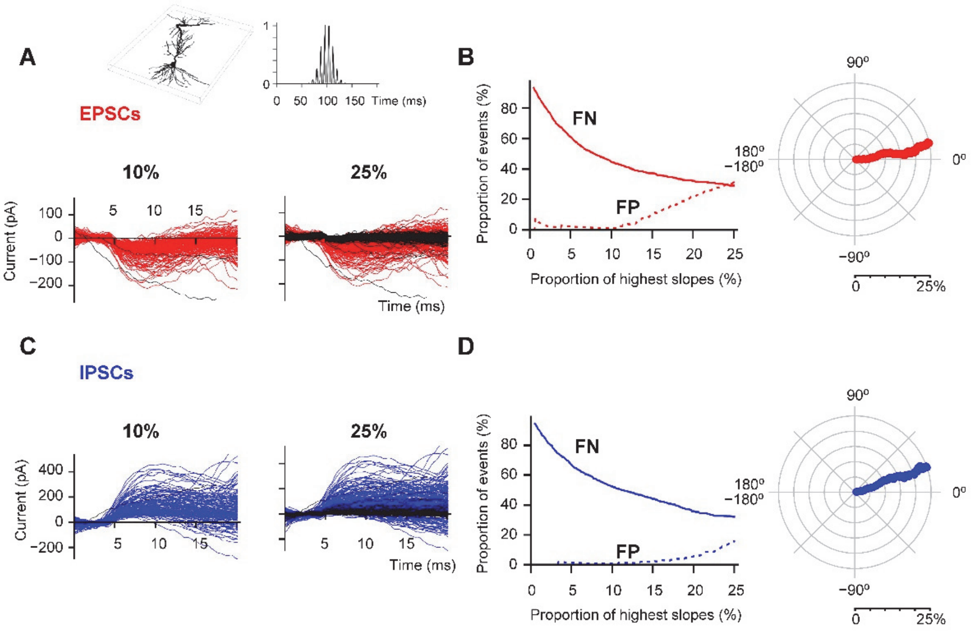

(A) Detection of EPSCs. Left, EPSCs with 10% largest derivative maxima. Right, EPSCs with 25% largest values. Red traces represent valid events, black traces indicate false-positive events. Inset, morphological reconstruction of *in vivo* labeled CA1 pyramidal neuron used for simulations (left) and probability density function used to simulate EPSCs associated to SWRs (right). EPSCs were generated randomly (75% according to the depicted probability density function, 25% according to an equal distribution).

(B) Left, fraction of false-negative (continuous line, FN) and false-positive events (dashed line, FP), plotted against percentage of largest derivative peaks. Right, polar plot of mean phase angle of detected events, plotted against percentage of largest derivative peaks. A phase of 0° corresponds to peak of individual ripple cycles. Note that the phase of the synaptic events is reliably detected by the algorithm.

(C and D) Similar analysis as in (A) and (B), but for IPSCs. Synaptic peak conductance 0.75 nS (EPSCs) and 1.5 nS (IPSCs).

EXPERIMENTAL PROCEDURES

Animal preparation and surgery

All experiments were carried out in strict accordance with institutional, national, and European guidelines for animal experimentation. Protocols were approved by the Bundesministerium für Wissenschaft und Forschung of Austria (BMWF-66.018/0008-II/3b/2010, BMWFW-66.018/0007-WF/II/3b/2014). Male or female 5- to 8-week-old C57Bl/6J mice were used in this study. Animals were maintained under light (7 am–7 pm) and dark cycle (7 pm–7 am) conditions, and housed under single animal per cage conditions until the day of experiment. Experiments were performed from 10 am–7 pm.

Mice were initially anesthetized by i.p. injection of 100 mg kg^{-1} ketamine (Intervet) and 10 mg kg^{-1} xylazine (Graeub), before mounting in a stereotaxic frame (David Kopf Instruments), in which the head of the animal was fixed with a pair of ear bars and a perpendicular tooth bar. The position of the animal's head was adjusted to assure that the micromanipulator axis was precisely perpendicular to the transversal plane. Mice were supplied with 100% oxygen through a ventilation mask and respiratory and cardiovascular functions were continuously monitored by measuring arterial oxygen saturation level and pulse frequency using an oximeter system (PulseSense^{TM} Vet, Medair). Typically, arterial oxygen saturation was >98% and heart rate was 250–320 beats min^{-1}. Body temperature was continuously monitored by a rectal thermometer and maintained at 37 ± 0.5°C by placing the animal on a custom-made heating pad.

For surgery, the skull of the animal was exposed and dried. A small craniotomy (~2 mm diameter) was made on the right hemisphere to target the dorsal hippocampus according to stereotaxic coordinates at AP ≈ 1.8 mm and L ≈ 1.5 mm (Paxinos and Franklin, 2001; AP, anterioposterior from bregma; L, lateral from midline). Extreme care was taken to minimize bleeding and to avoid rupturing of the *dura mater* at this stage. In addition, up to 4 fixation holes (~1 mm diameter) were drilled into the skull for mounting (2 frontal, 2 posterior). A custom-made fixation ring (GFK fiberglass, R&G Faserverbundwerkstoffe) was attached tightly to the skull via microscrews inserted into the fixation holes and additionally fixed using dental cement (Paladur; Heraeus). Ear and tooth bars were removed after the dental cement was fully cured. Subsequently, within the craniotomy window, the *dura mater* was carefully cut and removed using iridectomy scissors and Dumont #5 forceps (FST). The exposed cortical surface was superfused with HEPES-buffered extracellular solution (135 mM NaCl, 3.5 mM KCl,

1.8 mM $CaCl_2$, 1 mM $MgCl_2$, and 5 mM HEPES; pH = 7.28 with NaOH). Afterwards, the mouse was injected with 0.1 mg kg^{-1} buprenorphine subcutaneously for analgesia and placed on a flat rotatable sitting pad to allow for recovery from anesthesia. Thus, the mouse was stably head-fixed via the fixation ring only, without any further physical constraints.

Upon recovery from anesthesia, typically after 0.8–1.5 h, all sensors attached to the animal were removed. Mice were allowed to steadily adapt to the head-fixed configuration. Under head-restraint conditions, mice were able to groom, rest, and run. Vigilance of the animals was judged by high muscle tone, active movement of whiskers, tail, and limbs, postural reactions, and locomotive patterns. Typically, after 2–4 hours habituation, mice were able to rest quietly for periods of 10–15 minutes, which allowed us to perform stable whole-cell patch-clamp recordings.

Whole-cell patch-clamp recordings from CA1 pyramidal neurons in awake mice

Patch pipettes were fabricated with a Brown-Flaming micropipette puller (P-1000; Sutter Instrument), using 1.0 mm / 0.5 mm (outer diameter / inner diameter) borosilicate glass tubing (Hilgenberg) and had tip resistance values of 4–6 MΩ. For voltage-clamp recordings, pipettes were filled with a Cs^+-based intracellular solution, containing 130 mM Cs-methanesulfonate, 2 mM KCl, 10 mM EGTA, 2 mM $MgCl_2$, 2 mM Na_2ATP, 10 mM HEPES, 5 mM QX-314, and 3 mg ml^{-1} biocytin (pH adjusted to 7.28 with CsOH, 290–300 mOsm). A reference electrode (Ag–AgCl) was placed on the skull near the craniotomy window. The cortical surface was immersed with HEPES-buffered extracellular solution. The pyramidal cell layer of the dorsal hippocampus was targeted using age-corrected stereotaxic coordinates (AP: 1.8–2.0 mm; L: 1.5–2.0 mm; and DV: 1.1–1.4 mm; DV: dorsal-ventral from cortical surface; Paxinos and Franklin, 2001). Patch pipettes were gently advanced from the craniotomy window perpendicular to the cortical surface with positive pressure (~300 mbar) applied to the pipette lumen to avoid tip plugging, until ~200 µm above the target area. Subsequently, pressure was reduced to ~15 mbar. Next, the tight-seal cell-attached configuration was reached in the voltage-clamp mode, with pipette holding potential set at −70 mV to minimize holding current (typically 0 to –5 pA). Finally, the whole-cell patch-clamp recording configuration was obtained, monitored by changes in current amplitudes in response to a 10-mV test pulse (Margrie et al., 2002; Lee et al., 2006; Lee et al., 2009; Pernía-Andrade and Jonas, 2014). The number of recording attempts was kept to a minimum

(1–3 attempts per mouse) to avoid tissue damage and spurious biocytin labeling. Only cells with initial seal resistance >5 GΩ were included in this study. After completion of the experiment, the integrity of the seal was verified by the formation of an outside-out patch during pipette withdrawal. Maximal care was taken to minimize the series resistance (R_s) during recording, which was 17 ± 1 MΩ in the present data set (range: 10–27 MΩ). R_s was carefully monitored throughout the recording session using 20-ms, 10-mV hyperpolarizing test pulses applied at ~1 min intervals. EPSCs and IPSCs were recorded in the voltage-clamp configuration with the same cell held at either −70 mV or +10 mV, respectively, with alternating order. Corresponding holding currents were, on average, −283.6 ± 50.4 pA and 290.0 ± 29.5 pA, respectively. Membrane potential values reported were not corrected for liquid junction potentials.

Local field potential recording in awake mice

LFP recording pipettes were fabricated from 1.0 mm / 0.5 mm (outer diameter / inner diameter) borosilicate glass tubing (Hilgenberg) and had open-tip resistance values of 1–3 MΩ. LFP pipettes were filled with HEPES-buffered extracellular solution containing 3 mg ml^{-1} biocytin. The LFP pipettes were carefully inserted from the same craniotomy as the patch pipettes, at a 25° oblique angle, in the AP direction, targeting the CA1 pyramidal cell layer of the dorsal hippocampus (AP: 1.8–2.0 mm, L: 1.8–2.0 mm, DV: 1.1–1.3 mm). Positive pressure (50–80 mbar) was applied to avoid pipette plugging. The location of LFP pipette was visualized by histology of the pipette track and by post-hoc biocytin labeling (Pernía-Andrade and Jonas, 2014). To unequivocally determine pipette location, only a single LFP pipette was inserted per animal. To correlate whole-cell and LFP signals in the same brain region, we tried to minimize the distance between pipette tips; in the present recordings, the average distance was 348 ± 31 µm (range 181–710 µm).

Biocytin labeling

For analysis of CA1 neuron morphology after recording, brains were fixed for >24 h in 2.5% paraformaldehyde, 1.25% glutaraldehyde, and 15% saturated picric acid in 100 mM phosphate buffer (PB; pH 7.35). The hemisphere containing the recorded cell was cut into 200-µm thick parasagittal slices. After fixation, slices were washed, incubated in 2% hydrogen peroxide, and shock-frozen in liquid nitrogen. Subsequently, the tissue was treated with PB containing 1% avidin–biotinylated horseradish

peroxidase complex (ABC; Vector Laboratories) overnight at 4ºC. Excess ABC was removed by several rinses with PB, before development with 0.05% 3,3'-diaminobenzidine tetrahydrochloride and 0.01% hydrogen peroxide. Subsequently, slices were rinsed in PB several times and embedded in Mowiol (Roth). 11 out of 17 neurons were morphologically recovered; all showed the characteristics of CA1 pyramidal cells.

Optogenetics

PV^+ interneurons were inhibited or stimulated optogenetically. PV-Cre mice were kindly provided by Sylvia Arber (Hippenmeyer et al., 2005; RRID: IMSR_JAX:008069). Recombinant adeno-associated virus serotype 5 containing ArchT (rAAV5-CAG-FLEX-ArchT-GFP; Addgene # 23807) was obtained from University of North Carolina Vector Core (Han et al., 2011). Virus was injected into 4-week-old mice into the dorsal hippocampal CA1 region (AP: 1.8–2.2 mm; L: 1.6–2.0 mm; and DV: 1.0–1.4 mm; 9 injection sites total; 200 nl for each site; $8.5 \cdot 10^{11}$ vg ml^{-1}). Recombinant adeno-associated virus serotype 1 containing ChR2(H134R) (rAAV1-EF1α–DIO–ChR2(H134R)-mCherry; Addgene # 20297) was provided by UPenn Vector Core. Virus was injected into 4-week-old mice into the dorsal hippocampal CA1 region (AP: 1.9 mm; L: 1.6 mm; and DV: 1.0–1.4 mm; 3 injection sites total; 200 nl for each site; $4.5 \cdot 10^{13}$ vg ml^{-1}). After 3–4 weeks, mice were used for combined whole-cell and LFP recordings. An optical fiber (diameter 200 µm; Thor labs) was inserted (AP: 2.0 mm; L: 1.8 mm; and DV: 1.0–1.1 mm). Light pulses were applied using diode-pumped solid state lasers (594 nm, Omicron; and 473 nm, CNI). Laser intensity was set to reach a mean power of 27.6 mW and 35.6 mW, respectively, at the tip of the optical fiber. The reversal potential of IPSCs evoked by optogenetic stimulation of PV^+ interneurons was −65.8 ± 3.3 mV (4 whole-cell recordings), suggesting that excitation and inhibition can be accurately distinguished with the holding potentials used (**Figure S6**). To examine localization and specificity of viral infection, AAV-injected mice were transcardially perfused, using 4% paraformaldehyde in 100 mM phosphate buffer for fixation (PB; pH 7.35). For double labeling, a rabbit antibody against parvalbumin was used (PV-28; 1:1000; Swant; RRID: AB_2315235).

Data acquisition and analysis

Patch-clamp and LFP recordings were made with an EPC 10 Quadro amplifier (HEKA) with two independent channels. Both signals were low pass-filtered at 10 kHz (Bessel), sampled at 20 kHz, and stored using Patchmaster v2x60 software running on a PC under Windows XP. Before further data analysis, synaptic signals were additionally filtered using a digital 1 kHz low-pass Gaussian filter. Likewise, LFP signals were additionally filtered using a digital 500 Hz low-pass Gaussian filter. Data analyses were performed using Stimfit (version 0.13 or 0.14; Guzman et al., 2014), Spike2 (version 7.01; Cambridge Electronic Design), Igor Pro (version 6.22; WaveMetrics), Sigviewer 0.5x (biosig.sourceforge.net), and custom-made scripts in Matlab (R2013a, Mathworks) or Octave (www.gnu.org). Frequency–time analysis was performed with short-time Fourier transform, using 204.8-ms time widows with 50% overlap. Spontaneous EPSCs or IPSCs (**Figure S1**) were detected using a template fit algorithm (Jonas et al., 1993; Pernía-Andrade et al., 2012). Cells were only included in the analysis if they met the following criteria: (1) series resistance < 30 MΩ, (2) stability of series resistance during recording, and (3) recording from several alternating epochs of EPSCs and IPSCs (at least 3 per condition).

Sharp wave-ripple detection and triggered analysis of postsynaptic currents

SWR detection was implemented using a root mean square (RMS) power threshold-based algorithm, as previously described (Csicsvari et al., 1999). Wide band LFP signals were first filtered between 100–250 Hz (FFT filter of Matlab), and the RMS power was integrated over a 10-ms sliding window. SWR events were detected when RMS peaks exceeded a threshold of mean + 4 x the standard deviation of baseline activity (SD) in a 30-s recording episode. A qualified RMS peak was defined in the center of an SWR event. The start and end points of an SWR event were taken as the first and last crossing with a mean + 1 x SD line around the peak. Individual ripple cycles were identified as events above the mean + 1 x SD line. All SWR events were visually confirmed. For SWR-triggered analysis of EPSCs or IPSCs, the center of the SWR event was taken as reference (time zero), and a ±200 ms data window centered on this reference time, including both LFP and postsynaptic current traces, was exported for subsequent analysis.

±200-ms epochs of SWR-triggered postsynaptic currents were baseline-subtracted, aligned to the maximal positive ripple deflection in the band pass-filtered

LFP, and averaged separately for excitatory or inhibitory conditions, respectively, within one cell (49 ± 21 SWRs per cell). Excitatory and inhibitory postsynaptic conductances were calculated as $G_{E/I} = I_{E/I} / (V_h - E_{rev,\ E/I})$, where $G_{E/I}$ is the SWR-associated conductance (EPSG or IPSG), $I_{E/I}$ is the corresponding current (EPSC or IPSC), V_h is the holding potential (–70 mV and +10 mV, respectively), and $E_{rev,\ E/I}$ is the reversal potential. Assuming a bicarbonate concentration of 25 mM (intra- and extracellularly) and a P_{HCO3} / P_{Cl} of 0.18 (Bormann et al., 1987), the reversal potential of $GABA_A$ receptor-mediated conductances was estimated as −70.8 mV. Kinetics of SWR-associated EPSCs or IPSCs were measured using Stimfit (Guzman et al., 2014). To quantify the relationship between amplitude of SWRs and the corresponding magnitude of SWR-associated synaptic conductances, we calculated the maximal positive ripple amplitude in the band pass-filtered LFP and the peak of the associated EPSG or IPSG in each SWR event. To increase the reliability of this analysis, we only considered cells in which small SWR events (4 SD < RMS power < 7 SD) coexisted with large SWR events (RMS power ≥ 7 SD).

Derivative-based analysis of postsynaptic currents

To quantitatively examine the temporal structure and relationship between SWR-associated synaptic conductances and ripple oscillations *in vivo*, we used a first derivative-based detection method (Maier et al., 2011). The method exploits the characteristic kinetic property of synaptic currents, which is a fast rise followed by a slower decay. For derivative analysis, we first processed SWR-associated EPSCs or IPSCs with a 0.5–400-Hz band pass filter (zero-phase 2nd order Butterworth; using the filtfilt function of Matlab). Next, we calculated the first derivatives of the resulting traces, and marked their extrema in both upward and downward directions within a ±50 ms window around a SWR maximum. Finally, we detected EPSC onsets as derivative minima, and IPSC onsets as derivative maxima. To account for variability in the kinetic properties of synaptic currents, we analyzed different subsets of data representing the largest derivative peaks (Maier et al., 2011). The values of computed parameters (phase relations, IPSC-triggered LFP averages) remained consistent over a wide range of percentiles examined (1–25%).

To quantitatively describe the temporal relationship between fast LFP oscillations and synaptic dynamics on a “ripple cycle-by-cycle” basis, we first computed the Hilbert transform of the band pass-filtered LFP signal (100–250 Hz, FFT filter;

English et al., 2014). Next, each EPSC or IPSC onset time point was assigned a Hilbert phase value, φ_j. Finally, the mean phase angle was obtained for each cell, and averaged across cells. EPSC/IPSC-triggered averages of SWRs were computed by averaging ±50-ms stretches of LFP recording centered at the times of 10% largest derivative minima (EPSC onset) or derivative maxima (IPSC onset). Extracted LFP recordings were filtered in 0.5–400 Hz (wide band) or 100–250 Hz (ripple band) for illustrative purposes.

Simulations

To quantitatively assess voltage-clamp and event detection errors, CA1 pyramidal neurons recorded *in vivo* were traced using a digital reconstruction system (Neurolucida 9.0; Microbrightfield) and 60 x / 1.4 numerical aperture (NA) or 100 x / 1.4 NA oil-immersion objectives. Only cells in which the dendritic tree of the postsynaptic cell appeared largely intact were included in the analysis. To estimate voltage-clamp errors, we simulated EPSCs and IPSCs in realistic cable models of CA1 pyramidal neurons. After reconstruction of dendritic and axonal morphology, Neurolucida data were imported into Neuron 7.4 (Carnevale and Hines, 2006). The number of segments per section was set according to the d_lambda rule, so that the length of each segment was < 3.3% of length constant λ at 1000 Hz. The integration time step was set to 5 µs. Cable parameters were R_i = 150 Ω cm, R_m = 30,000 Ω cm^2, and C_m = 0.8 µF cm^{-2}. Synaptic conductances were simulated as double exponential functions, with rise time constant τ_{rise} (0.2 ms), decay time constant τ_{decay} (2.5 ms for excitatory synapses, 7.5 ms for inhibitory synapses), and reversal potential E_{syn} (0 mV for excitatory synapses, −65 mV for inhibitory synapses). Voltage-clamp conditions were established inserting a SEClamp point process at the center of the soma, assuming a series resistance of 10 MΩ.

For simulation of voltage-clamp errors (Jonas et al., 1993; Major et al., 1994; Williams and Mitchell, 2008; **Figure S3–S5**), synaptic peak conductance was assumed as 1 nS. Synapses were sequentially placed on all dendritic segments, and somatic EPSCs and IPSCs were simulated as the clamp current (i) of the SEClamp point process. For simulation of detection errors (**Figure S7**), peak conductance amplitudes were assumed to have a coefficient of variation of 0.6; mean peak conductance was 0.75 nS for excitatory and 1.5 nS for inhibitory events. Excitatory synapses were placed on dendrites < 300 µm from the center of the soma, approximately corresponding to

Schaffer collateral input. Inhibitory synapses were placed on dendrites < 150 μm from the center of the soma, approximately corresponding to inputs from basket or bistratified cells. Synaptic events were either simulated with random time points (25% of events), or with SWR temporal structure as a series of Gaussian functions with 8 ms spacing, with standard deviation of 1 ms, and amplitudes defined by a Gaussian envelope function, with standard deviation of 12 ms (75% of events). Gaussian white noise was added to the data, and simulated traces were filtered before analysis, similar to the experimental traces (400 Hz; 2nd order Butterworth characteristics). Events were classified as true positive if the time difference between detected and the nearest original event was in the range ±1 ms (after correction for the delay between onset and steepest point in the rising phase of a simulated EPSC or IPSC).

Statistics

Data are presented as means ± standard error of the mean (SEM). Differences were examined for statistical significance using a non-parametric Wilcoxon signed-rank test; differences were considered statistically significant if $P < 0.05$. Circular uniformity in phase relationship analysis was examined with a Rayleigh test (Zar, 2010). Throughout the paper, * indicates $P < 0.05$, ** $P < 0.01$, and *** $P < 0.001$.

Supplemental References

Bormann, J., Hamill, O.P., and Sakmann, B. (1987). Mechanism of anion permeation through channels gated by glycine and γ-aminobutyric acid in mouse cultured spinal neurones. J. Physiol. *385*, 243–286.

Carnevale, N.T., and Hines, M.L. (2006). The Neuron Book. (Cambridge, UK: Cambridge University Press).

Guzman, S.J., Schlögl, A., and Schmidt-Hieber, C. (2014). Stimfit: quantifying electrophysiological data with Python. Front. Neuroinform. *8*, 16.

Han, X., Chow, B.Y., Zhou, H., Klapoetke, N.C., Chuong, A., Rajimehr, R., Yang, A., Baratta, M.V., Winkle, J., Desimone, R., and Boyden, E.S. (2011). A high-light sensitivity optical neural silencer: development and application to optogenetic control of non-human primate cortex. Front. Syst. Neurosci. *5*, 18.

Hippenmeyer, S., Vrieseling, E., Sigrist, M., Portmann, T., Laengle, C., Ladle, D.R., and Arber, S. (2005). A developmental switch in the response of DRG neurons to ETS transcription factor signaling. PLoS Biol. *3*, e159.

Jonas, P., Major, G., and Sakmann, B. (1993). Quantal components of unitary EPSCs at the mossy fibre synapse on CA3 pyramidal cells of rat hippocampus. J. Physiol. *472*, 615–663.

Paxinos, G., and Franklin, K.B.J. (2001). The mouse brain in stereotaxic coordinates, 2nd edition. (San Diego, California: Academic Press).

Pernía-Andrade, A.J., Goswami, S.P., Stickler, Y., Fröbe, U., Schlögl, A., and Jonas, P. (2012). A deconvolution-based method with high sensitivity and temporal resolution for detection of spontaneous synaptic currents in vitro and in vivo. Biophys. J. *103*, 1429–1439.

Pernía-Andrade, A.J., and Jonas, P. (2014). Theta-gamma-modulated synaptic currents in hippocampal granule cells in vivo define a mechanism for network oscillations. Neuron *81*, 140–152.

Zar, J.H. (2010). Biostatistical analysis, 5th edition. (Upper Saddle River, New Jersey: Prentice Hall).
