# Supplementary material for: Phase-Locked Inhibition, but Not Excitation, Underlies Hippocampal Ripple Oscillations in Awake Mice In Vivo
Source: Neuron. 2017 Jan 18;93(2):308–14. doi: 10.1016/j.neuron.2016.12.018 (PMC5263253; doi:10.1016/j.neuron.2016.12.018)
Supplement: Document S2. Article plus Supplemental Information [file mmc2.pdf]

Report

Neuron

Phase-Locked Inhibition, but Not Excitation, Underlies Hippocampal Ripple Oscillations in Awake Mice In Vivo

Highlights

- High-resolution synaptic current recording during sharp wave-ripples (SWRs) in vivo
- Inhibition dominates over excitation during SWRs in the hippocampal CA1 region
- Phasic inhibition, but not excitation, is phase-locked to individual ripple cycles
- PV^+ interneurons substantially contribute to SWR-associated inhibitory conductance

Authors

Jian Gan, Shih-ming Weng, Alejandro J. Pernía-Andrade, Jozsef Csicsvari, Peter Jonas

Correspondence

peter.jonas@ist.ac.at

In Brief

Using high-resolution whole-cell recording of postsynaptic current from CA1 pyramidal neurons in awake mice, Gan et al. find that during sharp wave-ripples, inhibition dominates over excitation, is phase-locked to individual ripple cycles, and is primarily mediated by PV^+ interneurons.

Gan et al., 2017, Neuron *93*, 308–314
January 18, 2017 © 2017 The Author(s). Published by Elsevier Inc.
http://dx.doi.org/10.1016/j.neuron.2016.12.018

Neuron
Report

Phase-Locked Inhibition, but Not Excitation, Underlies Hippocampal Ripple Oscillations in Awake Mice In Vivo

Jian Gan,[1] Shih-ming Weng,[1,2,3] Alejandro J. Pernía-Andrade,[1,4] Jozsef Csicsvari,[1] and Peter Jonas[1,5,*]
[1]IST Austria (Institute of Science and Technology Austria), Am Campus 1, A-3400 Klosterneuburg, Austria
[2]Present address: Department of Speech Language Pathology and Audiology, National Taipei University of Nursing and Health Science, Taipei 11219, Taiwan
[3]Present address: Center for Drug Evaluation, Taipei 11557, Taiwan
[4]Present address: University of Vienna, Dr. Bohr-Gasse 7, Vienna Biocenter, A-1030 Vienna, Austria
[5]Lead Contact
*Correspondence: peter.jonas@ist.ac.at
http://dx.doi.org/10.1016/j.neuron.2016.12.018

SUMMARY

Sharp wave-ripple (SWR) oscillations play a key role in memory consolidation during non-rapid eye movement sleep, immobility, and consummatory behavior. However, whether temporally modulated synaptic excitation or inhibition underlies the ripples is controversial. To address this question, we performed simultaneous recordings of excitatory and inhibitory postsynaptic currents (EPSCs and IPSCs) and local field potentials (LFPs) in the CA1 region of awake mice in vivo. During SWRs, inhibition dominated over excitation, with a peak conductance ratio of 4.1 ± 0.5. Furthermore, the amplitude of SWR-associated IPSCs was positively correlated with SWR magnitude, whereas that of EPSCs was not. Finally, phase analysis indicated that IPSCs were phase-locked to individual ripple cycles, whereas EPSCs were uniformly distributed in phase space. Optogenetic inhibition indicated that PV$^+$ interneurons provided a major contribution to SWR-associated IPSCs. Thus, phasic inhibition, but not excitation, shapes SWR oscillations in the hippocampal CA1 region in vivo.

INTRODUCTION

Sharp wave-ripple (SWR) oscillations in the hippocampal CA1 region play a key role in memory consolidation during non-rapid eye movement sleep, immobility, and consummatory behavior (Buzsáki et al., 1992; Ylinen et al., 1995; Girardeau et al., 2009; Jadhav et al., 2012; reviewed by Buzsáki, 2015). Despite their important network function, the underlying synaptic mechanisms remain unclear. Several mechanisms of ripple generation have been proposed, including phasic excitation from the CA3 region (Maier et al., 2011), phasic inhibition from GABAergic interneurons (English et al., 2014), and gap junction coupling between pyramidal neurons (Draguhn et al., 1998). Divergent results may derive from technical limitations of the experimental approaches used. Extracellular local field potential (LFP) recording in vivo (Ylinen et al., 1995; Stark et al., 2014) can be applied to networks in awake, behaving animals, but does not allow accurate dissection of dendritic excitation and perisomatic inhibition, which will produce similar sink-source patterns. Intracellular analysis allows precise analysis of synaptic potentials, currents, and spiking, but is largely restricted to in vitro preparations (Hájos et al., 2013; Schlingloff et al., 2014), or to the analysis of synaptic potentials in vivo with limited temporal resolution (English et al., 2014; Hulse et al., 2016). Understanding the synaptic mechanisms of SWR generation requires recording of synaptic currents in CA1 pyramidal neurons in vivo in awake animals under voltage-clamp conditions, where excitation and inhibition can be precisely dissected (Borg-Graham et al., 1998).

RESULTS

To probe the synaptic mechanisms of SWR oscillations, we performed simultaneous recordings of excitatory and inhibitory synaptic currents (EPSCs and IPSCs) and LFPs in the CA1 region of awake mice in vivo (Figure 1). Animals were head-fixed, but fully awake, showing behaviors of grooming, whisking, and moving. We first tested whether functional characteristics of SWRs measured near the pyramidal cell layer under our experimental conditions were identical to those in awake, freely moving animals (Ylinen et al., 1995; Figures 1A–1C). On average, SWRs occurred with a mean frequency of 0.15 ± 0.02 Hz (694 events in 17 mice) and had a mean duration of 69.4 ± 4.3 ms. Individual ripple cycles were generated at a mean frequency of 154.0 ± 1.6 Hz, and the average number of ripples per sharp wave was 10.8 ± 0.7. These properties of SWRs were very similar to those reported previously in awake, behaving rodents (Ylinen et al., 1995; reviewed by Buzsáki, 2015).

Next, we investigated the postsynaptic currents during SWR oscillations in CA1 pyramidal neurons in the voltage-clamp configuration (Figures 1D–1F). To obtain adequate resolution, several efforts were made to optimize voltage-clamp conditions and to rigorously assess possible errors. First, we minimized series resistance (R_s), which on average was 17 ± 1 MΩ in our

 © 2017 The Author(s). Published by Elsevier Inc.
This is an open access article under the CC BY license (http://creativecommons.org/licenses/by/4.0/).

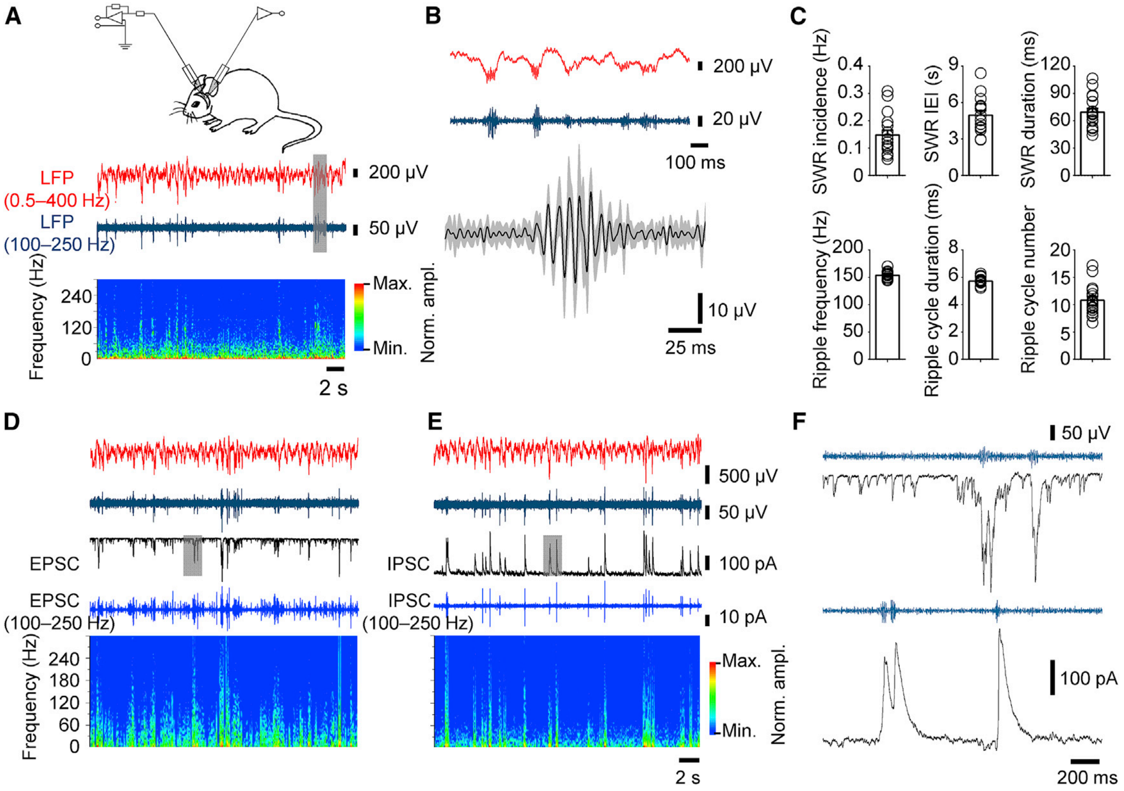

Figure 1. EPSCs and IPSCs Are Associated with SWRs in CA1 Pyramidal Neurons of Awake, Behaving Mice

(A) Top, wide band (red; 0.5–400 Hz) and band pass-filtered LFP trace (blue; 100–250 Hz). Bottom, frequency-time plot of the LFP with amplitude indicated by the color code (log scale; calibration bar on the right).

(B) Top, expanded view of wide band and band pass-filtered LFP trace (corresponding to the shaded area in A). Bottom, average of 26 SWRs (all SWRs recorded in one cell of a single awake mouse). Black line represents average trace; gray band indicates SEM. Individual SWRs were horizontally aligned to the maximal positive ripple deflection in the band pass-filtered LFP before averaging.

(C) Summary bar graphs of SWR properties. Top, SWR incidence, inter-event interval (IEI), and SWR duration. Bottom, ripple frequency, ripple cycle duration, and ripple cycle number per sharp wave. Bars represent mean ± SEM; circles indicate data from individual LFP recordings.

(D) Top, wide band (red; 0.5–400 Hz) and band pass-filtered LFP trace (blue; 100–250 Hz). Center, wide band EPSC trace (voltage clamp, black) and band pass-filtered EPSC trace (blue; 100–250 Hz). Holding potential was set to −70 mV to record EPSCs in isolation. Bottom, frequency-time plot of EPSC trace, with amplitude depicted by the color code (log scale; calibration bar in E, right).

(E) Similar plots as shown in (D), but for IPSCs. Holding potential was set to +10 mV to isolate IPSCs.

(F) Expanded view of EPSCs (top) and IPSCs (bottom) during SWRs (corresponding to the shaded areas in D and E).

Data in (D)–(F) were recorded from the same cell. Note that in the example traces IPSCs were more tightly correlated to the SWRs than EPSCs.

dataset (range, 10–27 MΩ; Margrie et al., 2002; Lee et al., 2006, 2009). Additionally, we used a cesium-based internal solution to enhance steady-state voltage control in distal dendrites (Williams and Mitchell, 2008). Second, we performed experimental tests for adequate voltage-clamp conditions. Measured spontaneous synaptic events showed a fast time course (Figure S1, available online), and extrapolation toward the limit of small R_s revealed even faster synaptic kinetics (Figure S2). Kinetic parameters and amplitude were only weakly correlated. Both results were consistent with adequate clamp conditions (Hestrin et al., 1990; Figure S2). Finally, we examined the extent of voltage-clamp errors for EPSCs and IPSCs in detailed cable models based on full reconstruction of in vivo-recorded CA1 pyramidal neurons (Major et al., 1994; Figures S3–S5). For proximal synapses, errors in peak amplitude, rise time, and decay time constant of somatically recorded EPSCs and IPSCs due to cable filtering were less than a factor of two (Figure S3), and corresponding postsynaptic voltage errors were <10 mV (Figure S4). Distributed pipette capacitance did not introduce additional errors (Figure S5). In conclusion, voltage-clamp errors were unavoidable, but appeared to be within an acceptable range under our experimental conditions.

Finally, we dissected EPSCs and IPSCs during SWRs by alternatingly setting the holding potential to either −70 mV (i.e., near the reversal potential of $GABA_A$ receptors; Figure S6) or +10 mV (i.e., near the reversal potential of AMPA- and NMDA-type glutamate receptors; Borg-Graham et al., 1998). Simultaneous recording of either EPSCs or IPSCs and the LFP revealed that the frequency of both excitatory and inhibitory events increased during SWRs (Figures 1D–1F). However, the generation of IPSCs (Figure 1E) appeared to be more tightly correlated to the SWRs than the generation of EPSCs (Figure 1D).

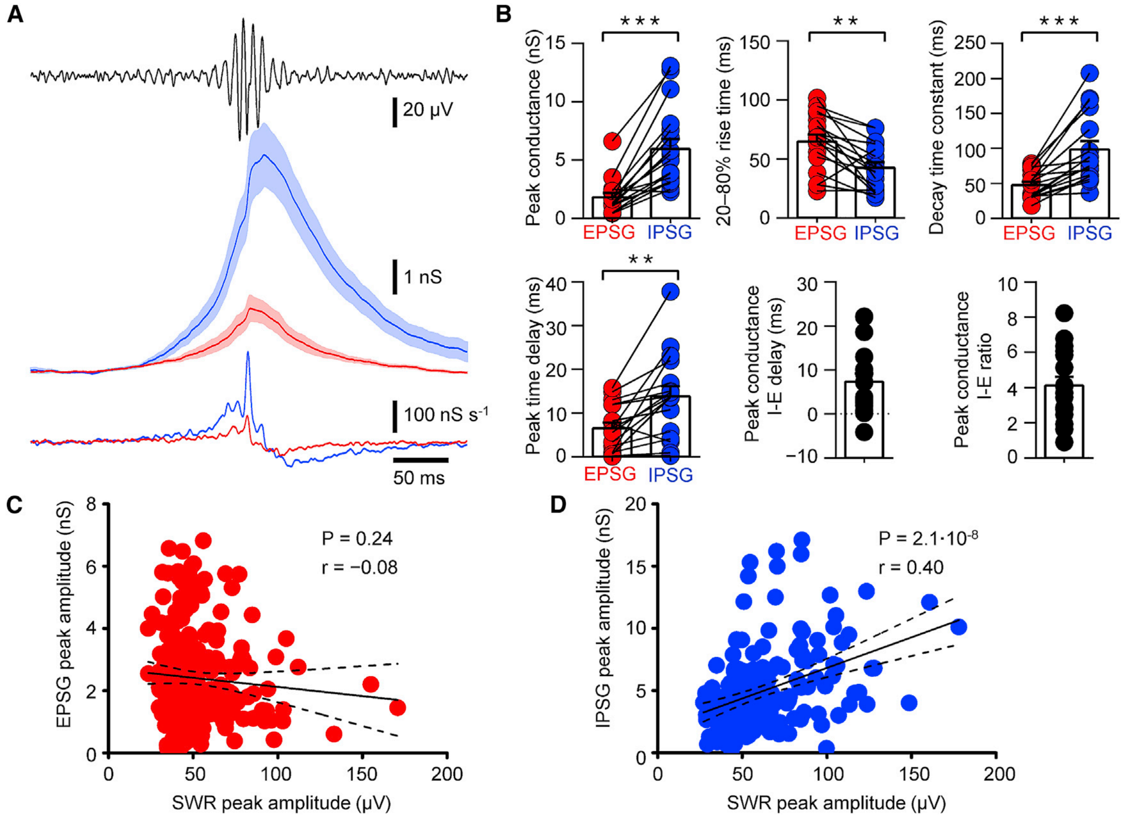

Figure 2. Inhibition Dominates over Excitation during SWRs and Correlates with Their Peak Amplitude

(A) SWR-triggered average of excitatory (red) and inhibitory (blue) conductance. Top, average band pass-filtered LFP trace (100–250 Hz); center, mean excitatory and inhibitory conductance; bottom, corresponding first derivatives. Red and blue lines indicate mean from 17 cells; light red and light blue areas represent SEM values. Excitatory and inhibitory conductance (EPSG and IPSG) was calculated from average EPSCs and IPSCs by division of currents by driving force. Note that the first derivative of IPSGs shows high-frequency oscillations.

(B) Summary bar graphs of SWR-triggered conductance properties. Top, peak conductance, 20%–80% rise time, and decay time constant of synaptic conductance. Bottom, delay between SWR center and peak of excitatory and inhibitory conductance, delay between peak of inhibitory and excitatory conductance, and corresponding conductance ratio. Bars represent mean ± SEM; circles indicate data from individual cells (red, excitatory events; blue, inhibitory events; black, data applying to both). Data from the same cell are connected by lines.

(C and D) Scatterplots of peak SWR-associated excitatory (C) and inhibitory postsynaptic peak conductance (D) against SWR peak amplitude. Each point represents an individual SWR. Continuous line represents the results of linear regression to the data points; dashed lines indicate 95% confidence intervals. There are a total of 238 SWRs in (C) and 185 SWRs in (D). Data are from ten cells in which SWR amplitude varied over a wide range. Note the lack of correlation between EPSG peak amplitude and SWR peak amplitude (C) and the highly significant positive correlation between IPSG peak amplitude and SWR peak amplitude (D).

To quantitatively analyze the contribution of excitation and inhibition to the generation of ripples, we performed SWR-triggered averaging of EPSCs and IPSCs (Figure 2). Synaptic currents were aligned to the maximal positive ripple deflection in the band pass-filtered LFP, averaged, and converted into synaptic conductances using the values of the driving force. SWR-triggered averaging revealed that the peak amplitude of the inhibitory conductance was markedly larger than that of the excitatory conductance (Figure 2A). On average, the SWR-associated peak conductance was 1.8 ± 0.4 nS for excitation and 6.0 ± 0.8 nS for inhibition (17 cells; $p < 0.0001$; Figures 2A and 2B), corresponding to an inhibition-to-excitation conductance ratio of 4.1 ± 0.5. In comparison to excitation, inhibition showed a faster rise (20%–80% rise time, 64.7 ± 5.9 ms for excitation and 42.5 ± 4.8 ms for inhibition; $p = 0.0021$; Figures 2A and 2B), but a slower decay (decay time constant, 47.6 ± 4.6 ms for excitation and 98.3 ± 12.1 ms for inhibition; $p < 0.0001$; Figures 2A and 2B). Furthermore, inhibition peaked at a slightly later time (time difference, 7.3 ± 1.9 ms; $p = 0.0005$). Thus, synaptic inhibition, rather than excitation (Maier et al., 2011), dominated during the generation of SWRs. Furthermore, excitation and inhibition showed different temporal profiles.

Previous studies revealed that the amplitude of SWRs varies from event to event by over an order of magnitude (Csicsvari et al., 2000). If either excitation or inhibition was responsible for ripple generation, we would expect that the relevant conductance would positively correlate with the ripple amplitude. To test this hypothesis, we plotted the peak amplitude of excitatory and inhibitory conductance in CA1 pyramidal neurons during individual SWRs against ripple amplitude (Figures 2C and 2D). For the excitatory conductance, peak conductance and ripple amplitude were not significantly correlated (linear correlation analysis,

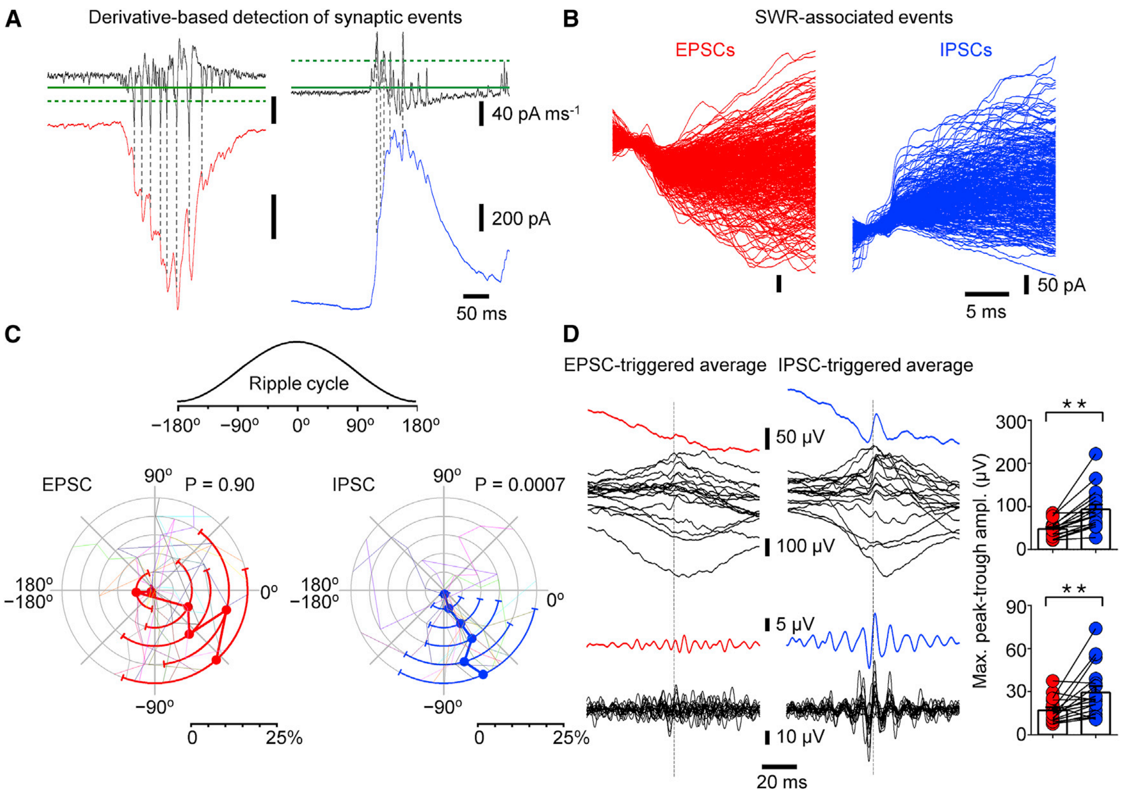

Figure 3. IPSCs, but Not EPSCs, Are Phase-Coupled to Ripple Oscillations

(A) Representative EPSCs at −70 mV (left) and IPSCs at +10 mV (right) associated with SWRs. Upper trace indicates first derivative of EPSC/IPSC traces; green horizontal lines represent 25% (continuous) and 10% (dashed) highest minima (left) or maxima (right) in the derivative trace. Gray vertical dashed lines indicate events detected using the maximal of the derivative trace. For details, see Experimental Procedures.

(B) Overlay of 372 detected EPSCs (left) and 278 detected IPSCs (right) during SWRs in a representative cell.

(C) Polar plot of mean phase of EPSC (left) and IPSC onset (right), as revealed by peak derivative detection. Concentric rings indicate results for different percentages of largest derivative peaks (1%, 5%, 10%, 15%, 20%, and 25%). Colored thin lines represent data from individual cells, red and blue symbols and lines indicate mean, and error bars plotted on top of concentric rings represent angular deviation. Left, analysis for EPSCs (red); right, similar data for IPSCs (blue). Data are from 17 cells. Inset on top illustrates phase angle conventions. Note that the angular deviations are much smaller for IPSCs than for EPSCs, consistent with differential phase locking. p values indicate results from Rayleigh test for 10% largest derivative peaks.

(D) EPSC/IPSC-triggered averaging of LFP traces. Left, analysis for EPSCs; center, similar analysis for IPSCs, for 10% largest derivative peaks in both cases. Top, wide band average LFPs (0.5–400 Hz). Bottom, band pass-filtered average LFPs (100–250 Hz). Black thin lines represent data from individual cells; colored lines indicate average (red, EPSC-triggered average; blue, IPSC-triggered average). Dashed vertical lines indicate EPSC and IPSC onset points, respectively. Right, summary bar graphs of maximal peak-to-trough amplitude of EPSC/IPSC-triggered average (red, EPSC; blue, IPSC; upper panels, wide band average LFPs [0.5–400 Hz]; bottom panels, band pass-filtered average LFPs [100–250 Hz]). Note that the band pass-filtered signal showed marked periodicity for IPSCs, but not for EPSCs.

Pearson's r = −0.08; 238 events; p = 0.24; Figure 2C). In contrast, for the inhibitory conductance, peak conductance and ripple amplitude exhibited a strong positive correlation (Pearson's r = 0.40; 185 events; p < 0.0001; Figure 2D). Thus, the magnitude of inhibition, but not that of excitation, was correlated with the amplitude of individual SWRs. Furthermore, excitation and inhibition were not strictly balanced during SWRs (Hulse et al., 2016).

To determine whether EPSCs and IPSCs were phase-locked to individual ripple cycles, we performed interleaved phase analysis for both EPSCs and IPSCs in CA1 pyramidal neurons (Figure 3). EPSC onset times were detected as minima of the first derivative of the membrane current at –70 mV, whereas IPSC onset times were determined as maxima of the first derivative at +10 mV (see Experimental Procedures; 3,566 and 3,180 events total for 25% largest derivative extrema; 17 cells; Figures 3A and 3B). Simulations using cable models based on cell reconstructions suggested that adequate event detection was achieved under these conditions (Figure S7). After computing the Hilbert transform of the band pass-filtered simultaneously recorded LFP signal (100–250 Hz; English et al., 2014), the onset times of EPSCs and IPSCs were individually assigned corresponding phase values, with 0° corresponding to the peak of a ripple. Whereas EPSCs were uniformly distributed (p = 0.90 for 10% largest derivative extrema; Rayleigh test; Figure 3C, left), IPSCs were significantly clustered in the late ascending phase of ripple cycles (p = 0.0007 for 10% largest derivative extrema; Rayleigh test; mean phase angle, −62.9° ± 49.6°, equivalent to 297.1°; Figure 3C, right). This conclusion was corroborated by reverse analysis using EPSC- or IPSC-triggered averaging of the LFP (Figure 3D). Synaptic event-triggered LFP averages

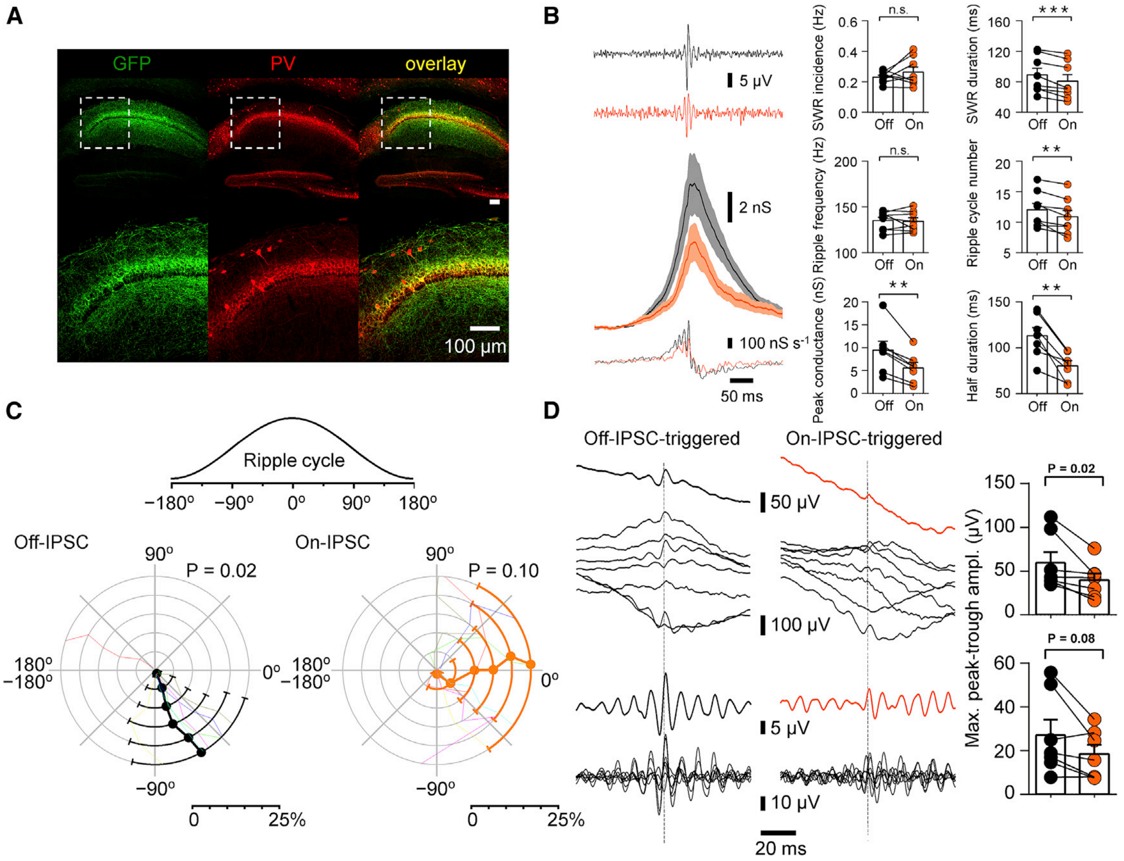

Figure 4. Optogenetic Suppression of PV⁺ Interneurons Reduces SWR-Associated Inhibitory Conductance and Disrupts Phase Preference of IPSCs

(A) AAV infection leads to selective expression of ArchT-GFP in PV⁺ interneurons of the CA1 region. Maximum intensity projection of confocal stacks. Left, GFP fluorescence; center, immunoreactivity for PV; right, overlay. Note the high degree of co-localization.

(B) Left, SWR-triggered average of inhibitory conductance in control period (black) and during laser illumination (orange). Top, average band pass-filtered LFP trace (100–250 Hz); center, mean inhibitory conductance from seven cells; bottom, corresponding first derivative. Gray and light orange areas represent SEM values. Inhibitory conductance was calculated from average IPSCs dividing current by driving force. Note that the first derivative of IPSGs in the absence of light shows high-frequency oscillations. Right, summary bar graphs of SWR properties (top four graphs) and inhibitory conductance properties (bottom two graphs). Black, laser illumination off; orange, laser illumination on. Note that optogenetic inhibition of PV⁺ interneurons significantly changes the properties of SWRs in the LFP and substantially reduces amplitude and duration of the inhibitory conductance.

(C) Polar plot of mean phase of IPSC onset, as revealed by peak derivative detection. Concentric rings indicate results for different percentages of largest derivative peaks (1%, 5%, 10%, 15%, 20%, and 25%). Colored thin lines represent data from individual cells, black and orange symbols and lines indicate mean, and error bars plotted on top of concentric rings represent angular deviation. Left, analysis for epochs with laser illumination off (black); right, similar data for epochs with laser illumination on (orange). Data are from seven cells. Inset on top illustrates phase angle conventions. Note significant phase locking in the off periods ($p = 0.02$; left; similar to Figure 3C, right panel), but not in the on periods ($p = 0.10$; right). p values indicate results from Rayleigh test for 10% largest derivative peaks.

(D) IPSC-triggered averaging of LFP traces. Left, analysis for epochs with laser illumination off (black); right, similar data for epochs with laser illumination on (orange) for 10% largest derivative peaks in both cases. Top, wide band average LFPs (0.5–400 Hz). Bottom, band pass-filtered average LFPs (100–250 Hz). Black thin lines represent data from individual cells; thick lines indicate averages (black, off epochs; orange, on epochs). Dashed vertical lines indicate IPSC onset points. Right, summary bar graphs of maximal peak-to-trough amplitude of IPSC-triggered average (black, off epochs; orange, on epochs; upper panels, wide band average LFPs [0.5–400 Hz]; bottom panels, band pass-filtered average LFPs [100–250 Hz]). Note that the IPSC-triggered LFP signals showed marked periodicity without laser illumination, but not with laser illumination.

were weakly rhythmically modulated for EPSC onsets (Figure 3D, left), but strongly modulated for IPSC onsets (Figure 3D, right). The peak-to-trough amplitude of the triggered LFP average was significantly larger for IPSCs than for EPSCs ($p < 0.0001$ and 0.0038). Thus, phasic inhibition, but not excitation, was phase-locked to individual cycles of ripple oscillations.

As PV⁺ interneurons strongly fire during SWRs (Lapray et al., 2012; Varga et al., 2012; see Hájos et al., 2013; Schlingloff et al., 2014), they are possible candidates for the generation of the SWR-associated inhibitory conductance. To test this hypothesis, we examined the effects of optogenetic inhibition of PV⁺ interneurons (Figure 4). The inhibitory opsin Archaerhodopsin T (ArchT) was selectively expressed in PV⁺ interneurons in the CA1 region of the hippocampus (Figure 4A). Five-second laser pulses were alternated with 11 s off periods, and properties of SWRs and associated inhibitory postsynaptic conductance

were compared between on and off periods (Figure 4B). Optogenetic inhibition of PV^+ interneurons did not change the incidence of SWRs, but significantly reduced SWR duration (from 89.3 ± 8.5 ms to 81.0 ± 8.1 ms; 8 mice; p = 0.008; Figure 4B, right top). Furthermore, light pulses did not change the ripple frequency, but decreased the number of ripple cycles per SWR (from 12.1 ± 1.0 to 10.9 ± 1.0; p = 0.008; Figure 4B, right center). Finally, optogenetic inhibition of PV^+ interneurons markedly reduced the peak amplitude of the SWR-associated inhibitory conductance (from 9.5 ± 1.9 nS to 5.6 ± 1.2 nS; 7 cells; p = 0.016) and reduced its half-duration (Figure 4B, right bottom). In conclusion, PV^+ interneurons make a major contribution to the inhibitory conductance during SWRs. Furthermore, they control the duration of SWRs and the number of ripples in SWR complexes.

If PV^+ interneurons are primarily responsible for SWR generation, optogenetic inhibition of these interneurons might result in a disruption of phase locking of IPSCs. To test this hypothesis, we performed phase analysis and IPSC-triggered LFP averaging before and after optogenetic inhibition after adeno-associated virus-mediated expression of ArchT (Figures 4C and 4D). In the absence of light, IPSCs were phase-locked to the ascending phase of the ripple cycle (Figure 4C, left), similar to the results in uninfected control animals (Figure 3C, right). In contrast, in the presence of light, the phase preference was perturbed (Figure 4C, right). Furthermore, the peak-to-trough amplitude of IPSC-triggered LFP averages was markedly reduced (Figure 4D). Thus, PV^+ interneurons provide a major contribution to phase-locked IPSCs during individual ripple cycles.

DISCUSSION

The present paper provides a quantitative analysis of postsynaptic conductances during SWRs in hippocampal CA1 pyramidal neurons in awake, behaving mice in vivo. Our results indicate that during SWRs, (1) inhibition dominates over excitation; (2) phasic inhibition, but not excitation, is positively correlated with SWR amplitude; (3) phasic inhibition, but not excitation, is phase-locked to individual ripple cycles; and (4) PV^+ interneurons provide a major contribution to the SWR-associated inhibitory conductance. Our findings directly reveal the current generator underlying SWRs. This was possible because our experiments provided information about both phase and location of the underlying conductance. The onset of the inhibitory conductance occurred at a phase of $\sim -60^\circ$, corresponding to the ascending phase of a ripple. Furthermore, the conductance was likely to be perisomatic because it was mediated, to a large extent, by PV^+ interneurons (Hu et al., 2014). Thus, the inhibitory conductance represents a perisomatic current source, resulting in a positive deflection in the LFP near the pyramidal cell layer.

Furthermore, our results shed light on the function of the rhythm generator. They suggest a model in which SWRs are generated by a combination of tonic excitation from CA3 and phasic inhibition within CA1. In contrast, our results are inconsistent with models in which phasic excitation is relayed from CA3 (Maier et al., 2011; see Nakashiba et al., 2009; Middleton and McHugh, 2016). Furthermore, they are incompatible with a major contribution of gap junctions (Draguhn et al., 1998). Whether ripple generation involves feedback inhibition, mutual inhibition, or both remains to be determined (Stark et al., 2014). The temporal sequence of events, with action potentials in CA1 pyramidal neurons at the ripple trough (Csicsvari et al., 1999), PV^+ interneuron firing in the early ascending phase (Lapray et al., 2012; Varga et al., 2012), and onset of inhibitory conductance in the late ascending phase of the ripples (this paper), would be consistent with a feedback mechanism (Stark et al., 2014). On the other hand, one might expect that GABAergic interneurons receive tonic excitation during SWRs, similar to that in CA1 pyramidal neurons. This could activate mutual inhibition circuits, independent of feedback innervation.

Finally, our findings have implications for temporal coding of information in the hippocampal CA1 region. Importantly, they explain why CA1 pyramidal neurons in vivo fire at the ripple trough (Csicsvari et al., 1999). In a ripple model based on phasic inhibition, the descending phase of the ripple coincides with the decay of the inhibitory conductance. Pyramidal cells will fire at a point where inhibition has become minimal, which is near the trough in the LFP. In this scenario, fast decay of the inhibitory conductance in CA1 pyramidal neurons in vivo (Figure S1) will ensure temporally precise action potential generation in these neurons (Carter and Regehr, 2002). Precise spike timing, in turn, may be critically important for the preplay or replay of temporal activity sequences in cell assemblies (Skaggs and McNaughton, 1996) and for the consolidation of memory via spike timing-dependent plasticity in downstream neocortical target cells.

EXPERIMENTAL PROCEDURES

In vivo whole-cell patch-clamp recordings from CA1 pyramidal neurons and simultaneous LFP recordings were performed in head-fixed, fully awake mice. All experiments were carried out in strict accordance with institutional, national, and European guidelines for animal experimentation. Protocols were approved by the Bundesministerium für Wissenschaft und Forschung of Austria (BMWF-66.018/0008-II/3b/2010, BMWFW-66.018/0007-WF/II/3b/2014). PV^+ interneurons were optogenetically manipulated using adeno-associated virus. EPSCs and IPSCs were detected using template- or derivative-based detection methods. Simulations were performed on detailed cable models of biocytin-filled CA1 pyramidal neurons. For details, see Supplemental Information.

SUPPLEMENTAL INFORMATION

Supplemental Information includes Supplemental Experimental Procedures and seven figures and can be found with this article online at http://dx.doi.org/10.1016/j.neuron.2016.12.018.

AUTHOR CONTRIBUTIONS

J.G. planned experiments, performed experiments, and analyzed data, S.-m.W. performed initial experiments, A.J.P.-A. supervised initial experiments, J.C. and P.J. conceived the project, and P.J. performed simulations and wrote the paper. All authors jointly revised the paper.

ACKNOWLEDGMENTS

We thank Hua Hu and Xiaomin Zhang for critically reading the manuscript, Jose Guzman for help with neuron reconstruction, Pradeep Bhandari for support with perfusion, and György Buzsáki for sharing unpublished material. We thank Edward Boyden, Karl Deisseroth, James Wilson, University of North

Carolina Vector Core, and University of Pennsylvania Vector Core for providing viral vectors. We are extremely grateful to A. Schlögl for programming, F. Marr for technical assistance, E. Kramberger for manuscript editing, and T. Asenov (Machine Shop) for device construction. We also thank the Scientific Service Units (SSUs) of IST Austria (Machine Shop, Scientific Computing, and Preclinical Facility) for help. This project has received funding from the Fond zur Förderung der Wissenschaftlichen Forschung (P 24909-B24) and the European Research Council (ERC) under the European Union's seventh framework programme with grant agreement number 268548 (both to P.J.).

Received: September 7, 2015
Revised: August 8, 2016
Accepted: December 6, 2016
Published: December 29, 2016

REFERENCES

Borg-Graham, L.J., Monier, C., and Frégnac, Y. (1998). Visual input evokes transient and strong shunting inhibition in visual cortical neurons. Nature *393*, 369–373.

Buzsáki, G. (2015). Hippocampal sharp wave-ripple: a cognitive biomarker for episodic memory and planning. Hippocampus *25*, 1073–1188.

Buzsáki, G., Horváth, Z., Urioste, R., Hetke, J., and Wise, K. (1992). High-frequency network oscillation in the hippocampus. Science *256*, 1025–1027.

Carter, A.G., and Regehr, W.G. (2002). Quantal events shape cerebellar interneuron firing. Nat. Neurosci. *5*, 1309–1318.

Csicsvari, J., Hirase, H., Czurkó, A., Mamiya, A., and Buzsáki, G. (1999). Fast network oscillations in the hippocampal CA1 region of the behaving rat. J. Neurosci. *19*, RC20.

Csicsvari, J., Hirase, H., Mamiya, A., and Buzsáki, G. (2000). Ensemble patterns of hippocampal CA3-CA1 neurons during sharp wave-associated population events. Neuron *28*, 585–594.

Draguhn, A., Traub, R.D., Schmitz, D., and Jefferys, J.G. (1998). Electrical coupling underlies high-frequency oscillations in the hippocampus in vitro. Nature *394*, 189–192.

English, D.F., Peyrache, A., Stark, E., Roux, L., Vallentin, D., Long, M.A., and Buzsáki, G. (2014). Excitation and inhibition compete to control spiking during hippocampal ripples: intracellular study in behaving mice. J. Neurosci. *34*, 16509–16517.

Girardeau, G., Benchenane, K., Wiener, S.I., Buzsáki, G., and Zugaro, M.B. (2009). Selective suppression of hippocampal ripples impairs spatial memory. Nat. Neurosci. *12*, 1222–1223.

Hájos, N., Karlócai, M.R., Németh, B., Ulbert, I., Monyer, H., Szabó, G., Erdélyi, F., Freund, T.F., and Gulyás, A.I. (2013). Input-output features of anatomically identified CA3 neurons during hippocampal sharp wave/ripple oscillation in vitro. J. Neurosci. *33*, 11677–11691.

Hestrin, S., Nicoll, R.A., Perkel, D.J., and Sah, P. (1990). Analysis of excitatory synaptic action in pyramidal cells using whole-cell recording from rat hippocampal slices. J. Physiol. *422*, 203–225.

Hu, H., Gan, J., and Jonas, P. (2014). Interneurons. Fast-spiking, parvalbumin$^+$ GABAergic interneurons: from cellular design to microcircuit function. Science *345*, 1255263.

Hulse, B.K., Moreaux, L.C., Lubenov, E.V., and Siapas, A.G. (2016). Membrane potential dynamics of CA1 pyramidal neurons during hippocampal ripples in awake mice. Neuron *89*, 800–813.

Jadhav, S.P., Kemere, C., German, P.W., and Frank, L.M. (2012). Awake hippocampal sharp-wave ripples support spatial memory. Science *336*, 1454–1458.

Lapray, D., Lasztoczi, B., Lagler, M., Viney, T.J., Katona, L., Valenti, O., Hartwich, K., Borhegyi, Z., Somogyi, P., and Klausberger, T. (2012). Behavior-dependent specialization of identified hippocampal interneurons. Nat. Neurosci. *15*, 1265–1271.

Lee, A.K., Manns, I.D., Sakmann, B., and Brecht, M. (2006). Whole-cell recordings in freely moving rats. Neuron *51*, 399–407.

Lee, A.K., Epsztein, J., and Brecht, M. (2009). Head-anchored whole-cell recordings in freely moving rats. Nat. Protoc. *4*, 385–392.

Maier, N., Tejero-Cantero, A., Dorrn, A.L., Winterer, J., Beed, P.S., Morris, G., Kempter, R., Poulet, J.F., Leibold, C., and Schmitz, D. (2011). Coherent phasic excitation during hippocampal ripples. Neuron *72*, 137–152.

Major, G., Larkman, A.U., Jonas, P., Sakmann, B., and Jack, J.J.B. (1994). Detailed passive cable models of whole-cell recorded CA3 pyramidal neurons in rat hippocampal slices. J. Neurosci. *14*, 4613–4638.

Margrie, T.W., Brecht, M., and Sakmann, B. (2002). In vivo, low-resistance, whole-cell recordings from neurons in the anaesthetized and awake mammalian brain. Pflugers Arch. *444*, 491–498.

Middleton, S.J., and McHugh, T.J. (2016). Silencing CA3 disrupts temporal coding in the CA1 ensemble. Nat. Neurosci. *19*, 945–951.

Nakashiba, T., Buhl, D.L., McHugh, T.J., and Tonegawa, S. (2009). Hippocampal CA3 output is crucial for ripple-associated reactivation and consolidation of memory. Neuron *62*, 781–787.

Schlingloff, D., Káli, S., Freund, T.F., Hájos, N., and Gulyás, A.I. (2014). Mechanisms of sharp wave initiation and ripple generation. J. Neurosci. *34*, 11385–11398.

Skaggs, W.E., and McNaughton, B.L. (1996). Replay of neuronal firing sequences in rat hippocampus during sleep following spatial experience. Science *271*, 1870–1873.

Stark, E., Roux, L., Eichler, R., Senzai, Y., Royer, S., and Buzsáki, G. (2014). Pyramidal cell-interneuron interactions underlie hippocampal ripple oscillations. Neuron *83*, 467–480.

Varga, C., Golshani, P., and Soltesz, I. (2012). Frequency-invariant temporal ordering of interneuronal discharges during hippocampal oscillations in awake mice. Proc. Natl. Acad. Sci. USA *109*, E2726–E2734.

Williams, S.R., and Mitchell, S.J. (2008). Direct measurement of somatic voltage clamp errors in central neurons. Nat. Neurosci. *11*, 790–798.

Ylinen, A., Bragin, A., Nádasdy, Z., Jandó, G., Szabó, I., Sik, A., and Buzsáki, G. (1995). Sharp wave-associated high-frequency oscillation (200 Hz) in the intact hippocampus: network and intracellular mechanisms. J. Neurosci. *15*, 30–46.

Neuron, Volume *93*

Supplemental Information

Phase-Locked Inhibition, but Not Excitation, Underlies Hippocampal Ripple Oscillations in Awake Mice In Vivo

Jian Gan, Shih-ming Weng, Alejandro J. Pernía-Andrade, Jozsef Csicsvari, and Peter Jonas

Figure S1. Properties of spontaneous EPSCs and IPSCs in CA1 pyramidal neurons *in vivo*, related to Figure 1.

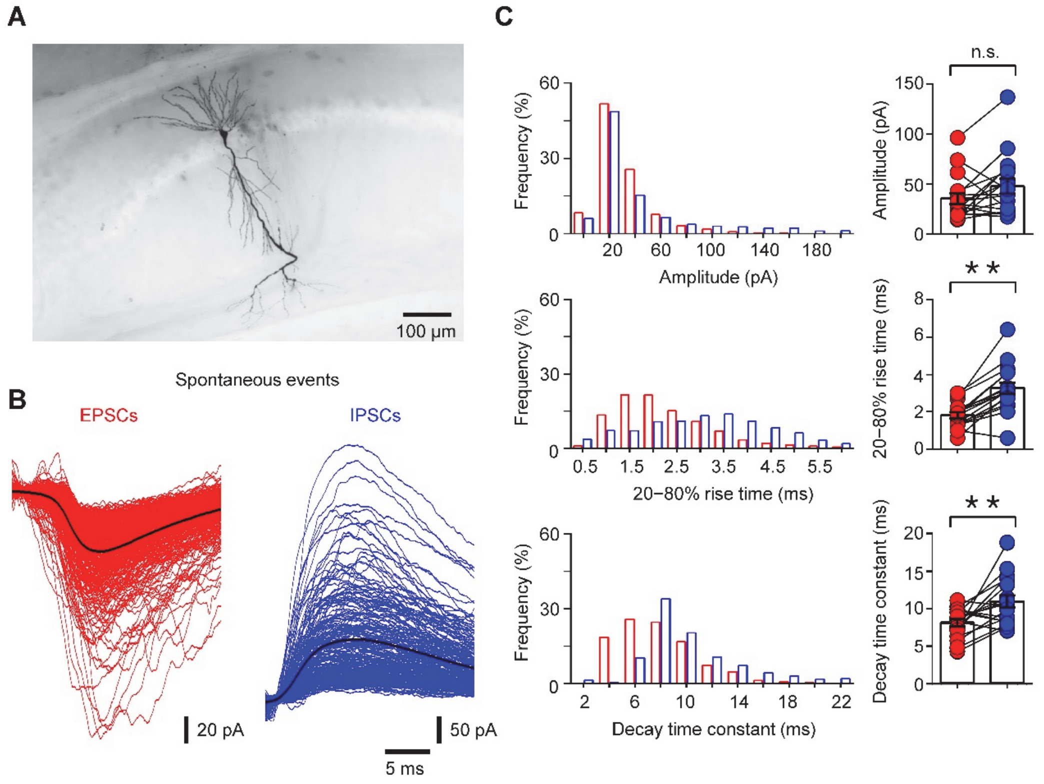

(A) Light micrograph of CA1 pyramidal neuron filled with biocytin during recording and visualized by 3,3'-diaminobenzidine.

(B) Superposition of 353 spontaneous EPSCs (left) and 322 spontaneous IPSCs (right). Data in A and B were obtained from the same cell. Holding potential was set to −70 mV and +10 mV to record EPSCs and IPSCs, respectively, in isolation.

(C) Peak amplitude, 20–80% rise time, and decay time constant of spontaneous EPSCs (red) and IPSCs (blue). Left, frequency distribution bar graphs of data from all cells (bin width: 20 pA, 0.5 ms, and 2 ms, respectively); right, summary bar graph obtained from individual experiments. Data from 17 cells.

Figure S2. Evidence for adequate voltage-clamp conditions in CA1 pyramidal neurons recorded *in vivo*, related to Figure 1.

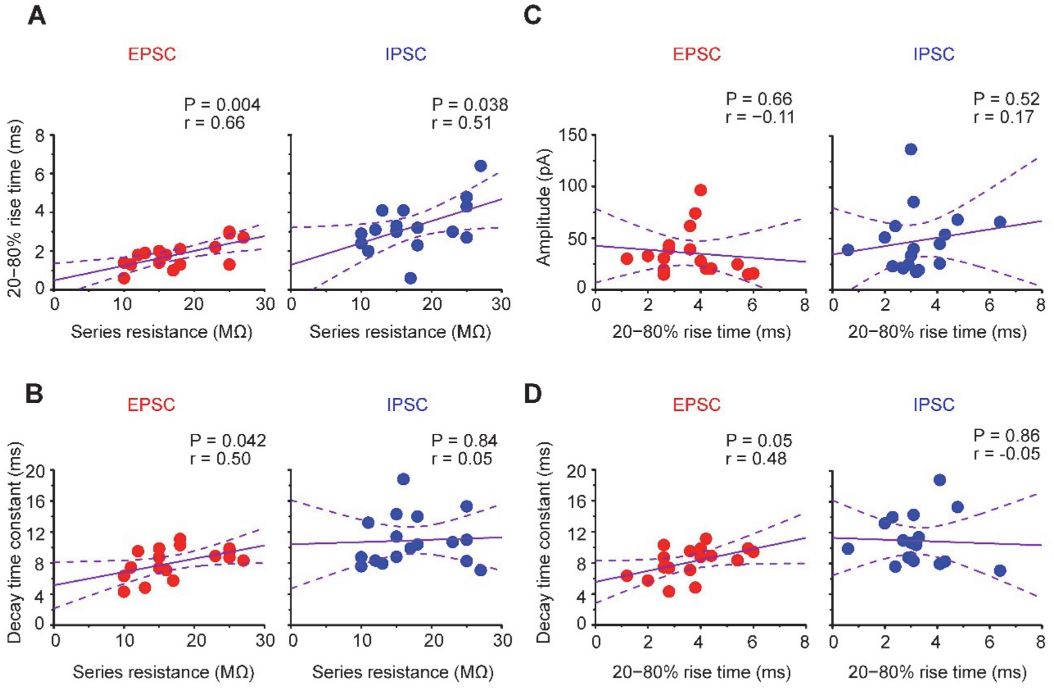

(A) Plot of 20–80% rise time against series resistance.

(B) Plot of decay time constant against series resistance. Extrapolation towards a series resistance of 0 may give information about the "true" kinetics of the synaptic currents.

(C) Scatter plot of peak amplitude versus 20–80% rise time. Left, EPSC data; right, IPSC data.

(D) Similar scatter plot of decay time constant versus rise time. Note lack of correlation or weak correlation (D, left), consistent with adequate voltage-clamp conditions (Hestrin et al., 1990).

Continuous lines represent the results of linear regression, dashed lines indicate the 95%-confidence intervals. Numbers indicate significance level P and corresponding Pearson's r. Data from 17 cells. In addition to analysis of population data, scatter plots were also analyzed for individual EPSCs and IPSCs on a cell-by-cell basis. Significant negative correlations between peak amplitude and 20–80% rise time were obtained in only 4 out of 17 cells. Furthermore, significant positive correlations between decay time constant and 20–80% rise time were obtained in only 2 out of 17 cells.

Figure S3. Simulation of space clamp errors in cable model based on reconstruction of *in vivo* labeled CA1 pyramidal neuron, related to Figures 1, 2, 3, and 4.

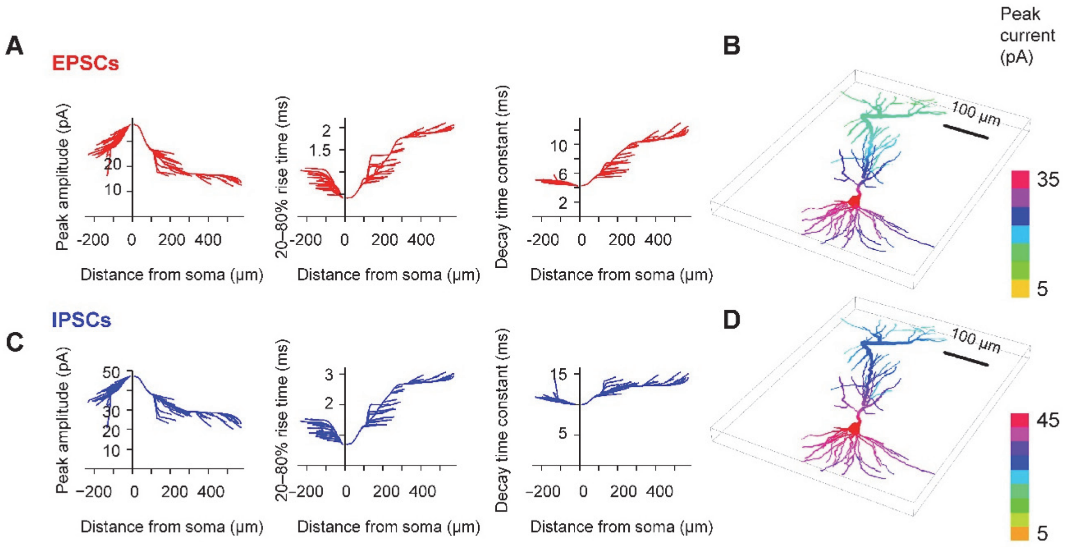

(A and B) Simulation of EPSCs (holding potential −70 mV, synaptic reversal potential 0 mV). Synapses were simulated at all possible dendritic locations, and somatic EPSCs were analyzed. (A) Plot of peak amplitude, 20–80% rise time, and decay time constant against distance (positive distance, apical dendrite; negative distance, basal dendrite). (B) Color-coded representation of the morphological properties of the CA1 pyramidal neuron, with color representing peak current amplitude of the somatic EPSC.

(C and D) Similar analysis as in (A) and (B), but for IPSCs (holding potential +10 mV, synaptic reversal potential −65 mV).

Synaptic peak conductance 1 nS; rise time constant 0.2 ms; decay time constant 2.5 ms (EPSCs) or 7.5 ms (IPSCs).

Figure S4. Simulation of postsynaptic voltage error during voltage-clamp experiments, related to Figures 1, 2, 3, and 4.

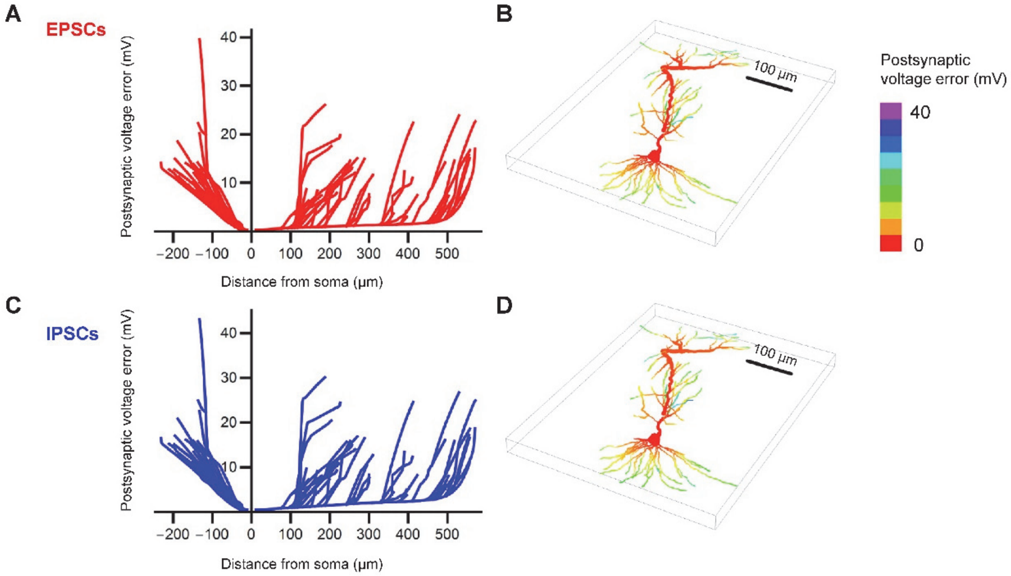

(A and B) Simulation of EPSCs (holding potential −70 mV, synaptic reversal potential 0 mV). Synapses were simulated at all possible dendritic locations, and somatic EPSCs were analyzed. (A) Plot of postsynaptic voltage change at the site of the synapse against distance (positive distance, apical dendrite; negative distance, basal dendrite). (B) Color-coded representation of the morphological properties of the CA1 pyramidal neuron, with color representing postsynaptic voltage error. Note that postsynaptic voltage error is small at proximal dendritic locations but increases near sealed ends.

(C and D) Similar analysis as in (A) and (B), but for IPSCs (holding potential +10 mV, synaptic reversal potential −65 mV).

Synaptic peak conductance 1 nS; rise time constant 0.2 ms; decay time constant 2.5 ms (EPSCs) or 7.5 ms (IPSCs).

Figure S5. Simulation of voltage-clamp errors with realistic pipette models, related to Figures 1, 2, 3, and 4.

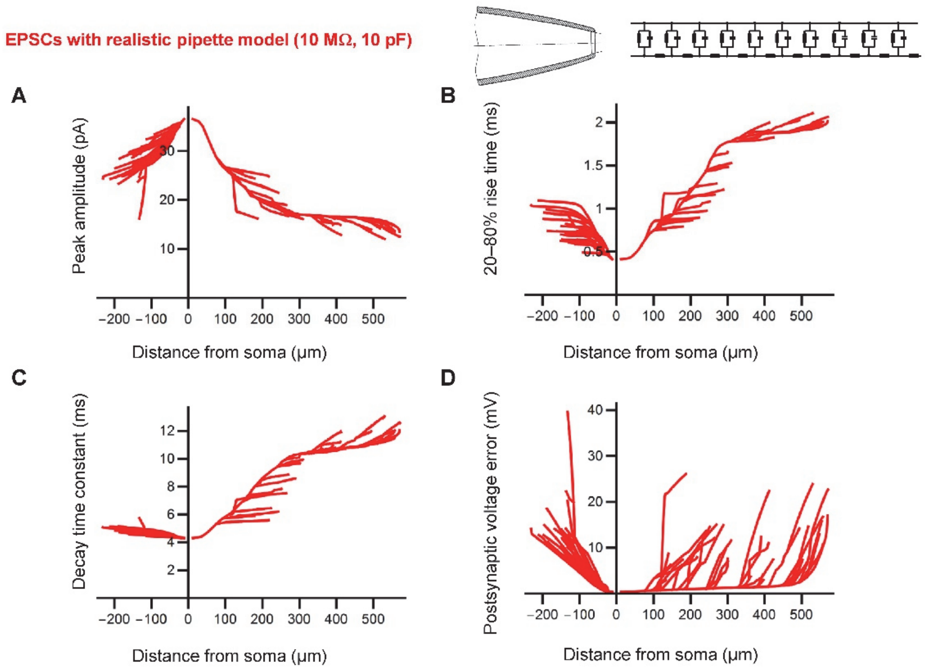

Simulation of EPSCs (holding potential −70 mV, synaptic reversal potential 0 mV). Synapses were simulated at all possible dendritic locations, and somatic EPSCs were analyzed. Plot of peak amplitude (A), 20–80% rise time (B), decay time constant (C), and postsynaptic voltage error (D) against distance (positive distance, apical dendrite; negative distance, basal dendrite). Pipette was implemented realistically as a multi-compartment structure; C_{pip} = 10 pF, R_s = 10 MΩ (scheme on top; 10 x 10 compartments). Results were almost identical to those without realistic pipette implementation (**Figure S3**A), indicating that errors introduced by the distributed nature of pipette capacitance and resistance were negligible.

Figure S6. Reversal potential of IPSCs in CA1 pyramidal neurons *in vivo*, evoked by optogenetic activation of PV⁺ interneurons, related to Figures 1, 2, 3, and 4.

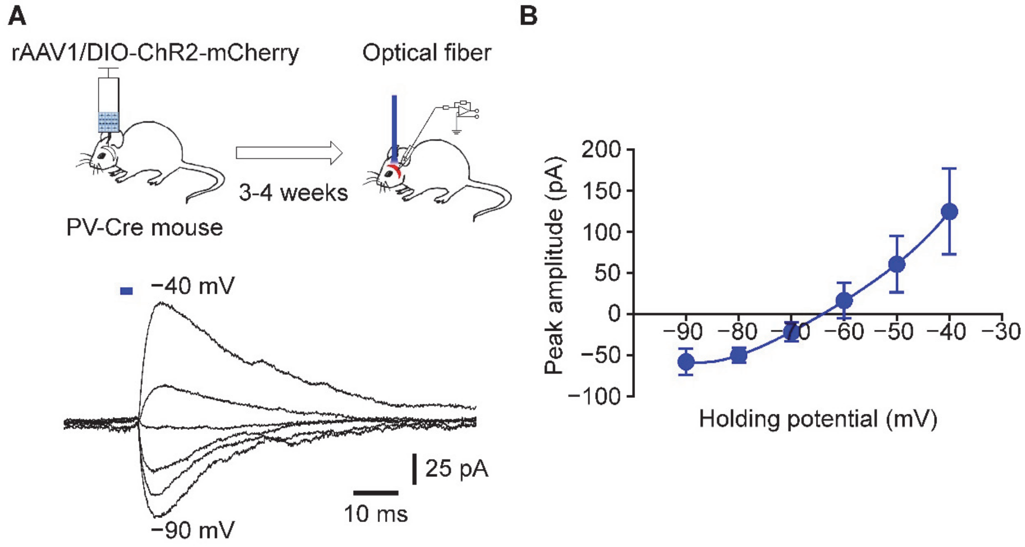

(A) Evoked IPSCs in CA1 pyramidal neurons at different holding potentials. Inset schematically shows whole-cell recording combined with optogenetic stimulation of PV⁺ interneurons.

(B) Current–voltage relation of IPSCs. Data points were fit with 4th-order polynomial function. Data from 4 neurons (in different mice).

Figure S7. Simulation of accuracy of derivative-based method for detection of EPSCs and IPSCs, related to Figures 3 and 4.

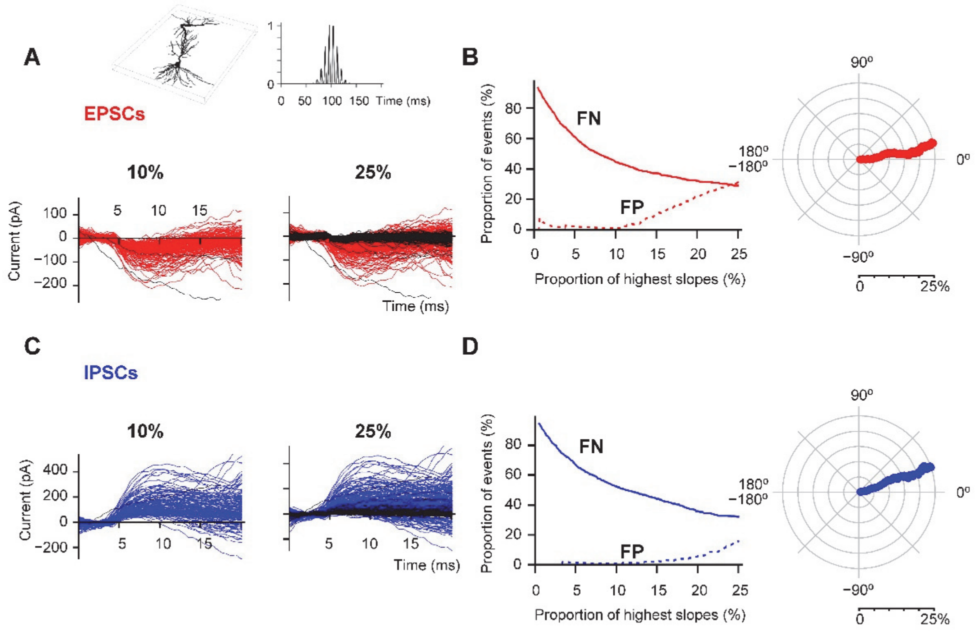

(A) Detection of EPSCs. Left, EPSCs with 10% largest derivative maxima. Right, EPSCs with 25% largest values. Red traces represent valid events, black traces indicate false-positive events. Inset, morphological reconstruction of *in vivo* labeled CA1 pyramidal neuron used for simulations (left) and probability density function used to simulate EPSCs associated to SWRs (right). EPSCs were generated randomly (75% according to the depicted probability density function, 25% according to an equal distribution).

(B) Left, fraction of false-negative (continuous line, FN) and false-positive events (dashed line, FP), plotted against percentage of largest derivative peaks. Right, polar plot of mean phase angle of detected events, plotted against percentage of largest derivative peaks. A phase of 0° corresponds to peak of individual ripple cycles. Note that the phase of the synaptic events is reliably detected by the algorithm.

(C and D) Similar analysis as in (A) and (B), but for IPSCs. Synaptic peak conductance 0.75 nS (EPSCs) and 1.5 nS (IPSCs).

EXPERIMENTAL PROCEDURES

Animal preparation and surgery

All experiments were carried out in strict accordance with institutional, national, and European guidelines for animal experimentation. Protocols were approved by the Bundesministerium für Wissenschaft und Forschung of Austria (BMWF-66.018/0008-II/3b/2010, BMWFW-66.018/0007-WF/II/3b/2014). Male or female 5- to 8-week-old C57Bl/6J mice were used in this study. Animals were maintained under light (7 am–7 pm) and dark cycle (7 pm–7 am) conditions, and housed under single animal per cage conditions until the day of experiment. Experiments were performed from 10 am–7 pm.

Mice were initially anesthetized by i.p. injection of 100 mg kg^{-1} ketamine (Intervet) and 10 mg kg^{-1} xylazine (Graeub), before mounting in a stereotaxic frame (David Kopf Instruments), in which the head of the animal was fixed with a pair of ear bars and a perpendicular tooth bar. The position of the animal's head was adjusted to assure that the micromanipulator axis was precisely perpendicular to the transversal plane. Mice were supplied with 100% oxygen through a ventilation mask and respiratory and cardiovascular functions were continuously monitored by measuring arterial oxygen saturation level and pulse frequency using an oximeter system (PulseSense^{TM} Vet, Medair). Typically, arterial oxygen saturation was >98% and heart rate was 250–320 beats min^{-1}. Body temperature was continuously monitored by a rectal thermometer and maintained at 37 ± 0.5°C by placing the animal on a custom-made heating pad.

For surgery, the skull of the animal was exposed and dried. A small craniotomy (~2 mm diameter) was made on the right hemisphere to target the dorsal hippocampus according to stereotaxic coordinates at AP ≈ 1.8 mm and L ≈ 1.5 mm (Paxinos and Franklin, 2001; AP, anterioposterior from bregma; L, lateral from midline). Extreme care was taken to minimize bleeding and to avoid rupturing of the *dura mater* at this stage. In addition, up to 4 fixation holes (~1 mm diameter) were drilled into the skull for mounting (2 frontal, 2 posterior). A custom-made fixation ring (GFK fiberglass, R&G Faserverbundwerkstoffe) was attached tightly to the skull via microscrews inserted into the fixation holes and additionally fixed using dental cement (Paladur; Heraeus). Ear and tooth bars were removed after the dental cement was fully cured. Subsequently, within the craniotomy window, the *dura mater* was carefully cut and removed using iridectomy scissors and Dumont #5 forceps (FST). The exposed cortical surface was superfused with HEPES-buffered extracellular solution (135 mM NaCl, 3.5 mM KCl,

1.8 mM $CaCl_2$, 1 mM $MgCl_2$, and 5 mM HEPES; pH = 7.28 with NaOH). Afterwards, the mouse was injected with 0.1 mg kg^{-1} buprenorphine subcutaneously for analgesia and placed on a flat rotatable sitting pad to allow for recovery from anesthesia. Thus, the mouse was stably head-fixed via the fixation ring only, without any further physical constraints.

Upon recovery from anesthesia, typically after 0.8–1.5 h, all sensors attached to the animal were removed. Mice were allowed to steadily adapt to the head-fixed configuration. Under head-restraint conditions, mice were able to groom, rest, and run. Vigilance of the animals was judged by high muscle tone, active movement of whiskers, tail, and limbs, postural reactions, and locomotive patterns. Typically, after 2–4 hours habituation, mice were able to rest quietly for periods of 10–15 minutes, which allowed us to perform stable whole-cell patch-clamp recordings.

Whole-cell patch-clamp recordings from CA1 pyramidal neurons in awake mice

Patch pipettes were fabricated with a Brown-Flaming micropipette puller (P-1000; Sutter Instrument), using 1.0 mm / 0.5 mm (outer diameter / inner diameter) borosilicate glass tubing (Hilgenberg) and had tip resistance values of 4–6 MΩ. For voltage-clamp recordings, pipettes were filled with a Cs^+-based intracellular solution, containing 130 mM Cs-methanesulfonate, 2 mM KCl, 10 mM EGTA, 2 mM $MgCl_2$, 2 mM Na_2ATP, 10 mM HEPES, 5 mM QX-314, and 3 mg ml^{-1} biocytin (pH adjusted to 7.28 with CsOH, 290–300 mOsm). A reference electrode (Ag–AgCl) was placed on the skull near the craniotomy window. The cortical surface was immersed with HEPES-buffered extracellular solution. The pyramidal cell layer of the dorsal hippocampus was targeted using age-corrected stereotaxic coordinates (AP: 1.8–2.0 mm; L: 1.5–2.0 mm; and DV: 1.1–1.4 mm; DV: dorsal-ventral from cortical surface; Paxinos and Franklin, 2001). Patch pipettes were gently advanced from the craniotomy window perpendicular to the cortical surface with positive pressure (~300 mbar) applied to the pipette lumen to avoid tip plugging, until ~200 µm above the target area. Subsequently, pressure was reduced to ~15 mbar. Next, the tight-seal cell-attached configuration was reached in the voltage-clamp mode, with pipette holding potential set at −70 mV to minimize holding current (typically 0 to –5 pA). Finally, the whole-cell patch-clamp recording configuration was obtained, monitored by changes in current amplitudes in response to a 10-mV test pulse (Margrie et al., 2002; Lee et al., 2006; Lee et al., 2009; Pernía-Andrade and Jonas, 2014). The number of recording attempts was kept to a minimum

(1–3 attempts per mouse) to avoid tissue damage and spurious biocytin labeling. Only cells with initial seal resistance >5 GΩ were included in this study. After completion of the experiment, the integrity of the seal was verified by the formation of an outside-out patch during pipette withdrawal. Maximal care was taken to minimize the series resistance (R_s) during recording, which was 17 ± 1 MΩ in the present data set (range: 10–27 MΩ). R_s was carefully monitored throughout the recording session using 20-ms, 10-mV hyperpolarizing test pulses applied at ~1 min intervals. EPSCs and IPSCs were recorded in the voltage-clamp configuration with the same cell held at either −70 mV or +10 mV, respectively, with alternating order. Corresponding holding currents were, on average, −283.6 ± 50.4 pA and 290.0 ± 29.5 pA, respectively. Membrane potential values reported were not corrected for liquid junction potentials.

Local field potential recording in awake mice

LFP recording pipettes were fabricated from 1.0 mm / 0.5 mm (outer diameter / inner diameter) borosilicate glass tubing (Hilgenberg) and had open-tip resistance values of 1–3 MΩ. LFP pipettes were filled with HEPES-buffered extracellular solution containing 3 mg ml^{-1} biocytin. The LFP pipettes were carefully inserted from the same craniotomy as the patch pipettes, at a 25° oblique angle, in the AP direction, targeting the CA1 pyramidal cell layer of the dorsal hippocampus (AP: 1.8–2.0 mm, L: 1.8–2.0 mm, DV: 1.1–1.3 mm). Positive pressure (50–80 mbar) was applied to avoid pipette plugging. The location of LFP pipette was visualized by histology of the pipette track and by post-hoc biocytin labeling (Pernía-Andrade and Jonas, 2014). To unequivocally determine pipette location, only a single LFP pipette was inserted per animal. To correlate whole-cell and LFP signals in the same brain region, we tried to minimize the distance between pipette tips; in the present recordings, the average distance was 348 ± 31 µm (range 181–710 µm).

Biocytin labeling

For analysis of CA1 neuron morphology after recording, brains were fixed for >24 h in 2.5% paraformaldehyde, 1.25% glutaraldehyde, and 15% saturated picric acid in 100 mM phosphate buffer (PB; pH 7.35). The hemisphere containing the recorded cell was cut into 200-µm thick parasagittal slices. After fixation, slices were washed, incubated in 2% hydrogen peroxide, and shock-frozen in liquid nitrogen. Subsequently, the tissue was treated with PB containing 1% avidin–biotinylated horseradish

peroxidase complex (ABC; Vector Laboratories) overnight at 4ºC. Excess ABC was removed by several rinses with PB, before development with 0.05% 3,3'-diaminobenzidine tetrahydrochloride and 0.01% hydrogen peroxide. Subsequently, slices were rinsed in PB several times and embedded in Mowiol (Roth). 11 out of 17 neurons were morphologically recovered; all showed the characteristics of CA1 pyramidal cells.

Optogenetics

PV^+ interneurons were inhibited or stimulated optogenetically. PV-Cre mice were kindly provided by Sylvia Arber (Hippenmeyer et al., 2005; RRID: IMSR_JAX:008069). Recombinant adeno-associated virus serotype 5 containing ArchT (rAAV5-CAG-FLEX-ArchT-GFP; Addgene # 23807) was obtained from University of North Carolina Vector Core (Han et al., 2011). Virus was injected into 4-week-old mice into the dorsal hippocampal CA1 region (AP: 1.8–2.2 mm; L: 1.6–2.0 mm; and DV: 1.0–1.4 mm; 9 injection sites total; 200 nl for each site; $8.5 \cdot 10^{11}$ vg ml^{-1}). Recombinant adeno-associated virus serotype 1 containing ChR2(H134R) (rAAV1-EF1α–DIO–ChR2(H134R)-mCherry; Addgene # 20297) was provided by UPenn Vector Core. Virus was injected into 4-week-old mice into the dorsal hippocampal CA1 region (AP: 1.9 mm; L: 1.6 mm; and DV: 1.0–1.4 mm; 3 injection sites total; 200 nl for each site; $4.5 \cdot 10^{13}$ vg ml^{-1}). After 3–4 weeks, mice were used for combined whole-cell and LFP recordings. An optical fiber (diameter 200 μm; Thor labs) was inserted (AP: 2.0 mm; L: 1.8 mm; and DV: 1.0–1.1 mm). Light pulses were applied using diode-pumped solid state lasers (594 nm, Omicron; and 473 nm, CNI). Laser intensity was set to reach a mean power of 27.6 mW and 35.6 mW, respectively, at the tip of the optical fiber. The reversal potential of IPSCs evoked by optogenetic stimulation of PV^+ interneurons was −65.8 ± 3.3 mV (4 whole-cell recordings), suggesting that excitation and inhibition can be accurately distinguished with the holding potentials used (**Figure S6**). To examine localization and specificity of viral infection, AAV-injected mice were transcardially perfused, using 4% paraformaldehyde in 100 mM phosphate buffer for fixation (PB; pH 7.35). For double labeling, a rabbit antibody against parvalbumin was used (PV-28; 1:1000; Swant; RRID: AB_2315235).

Data acquisition and analysis

Patch-clamp and LFP recordings were made with an EPC 10 Quadro amplifier (HEKA) with two independent channels. Both signals were low pass-filtered at 10 kHz (Bessel), sampled at 20 kHz, and stored using Patchmaster v2x60 software running on a PC under Windows XP. Before further data analysis, synaptic signals were additionally filtered using a digital 1 kHz low-pass Gaussian filter. Likewise, LFP signals were additionally filtered using a digital 500 Hz low-pass Gaussian filter. Data analyses were performed using Stimfit (version 0.13 or 0.14; Guzman et al., 2014), Spike2 (version 7.01; Cambridge Electronic Design), Igor Pro (version 6.22; WaveMetrics), Sigviewer 0.5x (biosig.sourceforge.net), and custom-made scripts in Matlab (R2013a, Mathworks) or Octave (www.gnu.org). Frequency–time analysis was performed with short-time Fourier transform, using 204.8-ms time widows with 50% overlap. Spontaneous EPSCs or IPSCs (**Figure S1**) were detected using a template fit algorithm (Jonas et al., 1993; Pernía-Andrade et al., 2012). Cells were only included in the analysis if they met the following criteria: (1) series resistance < 30 MΩ, (2) stability of series resistance during recording, and (3) recording from several alternating epochs of EPSCs and IPSCs (at least 3 per condition).

Sharp wave-ripple detection and triggered analysis of postsynaptic currents

SWR detection was implemented using a root mean square (RMS) power threshold-based algorithm, as previously described (Csicsvari et al., 1999). Wide band LFP signals were first filtered between 100–250 Hz (FFT filter of Matlab), and the RMS power was integrated over a 10-ms sliding window. SWR events were detected when RMS peaks exceeded a threshold of mean + 4 x the standard deviation of baseline activity (SD) in a 30-s recording episode. A qualified RMS peak was defined in the center of an SWR event. The start and end points of an SWR event were taken as the first and last crossing with a mean + 1 x SD line around the peak. Individual ripple cycles were identified as events above the mean + 1 x SD line. All SWR events were visually confirmed. For SWR-triggered analysis of EPSCs or IPSCs, the center of the SWR event was taken as reference (time zero), and a ±200 ms data window centered on this reference time, including both LFP and postsynaptic current traces, was exported for subsequent analysis.

±200-ms epochs of SWR-triggered postsynaptic currents were baseline-subtracted, aligned to the maximal positive ripple deflection in the band pass-filtered

LFP, and averaged separately for excitatory or inhibitory conditions, respectively, within one cell (49 ± 21 SWRs per cell). Excitatory and inhibitory postsynaptic conductances were calculated as $G_{E/I} = I_{E/I} / (V_h - E_{rev,\ E/I})$, where $G_{E/I}$ is the SWR-associated conductance (EPSG or IPSG), $I_{E/I}$ is the corresponding current (EPSC or IPSC), V_h is the holding potential (–70 mV and +10 mV, respectively), and $E_{rev,\ E/I}$ is the reversal potential. Assuming a bicarbonate concentration of 25 mM (intra- and extracellularly) and a P_{HCO3} / P_{Cl} of 0.18 (Bormann et al., 1987), the reversal potential of $GABA_A$ receptor-mediated conductances was estimated as −70.8 mV. Kinetics of SWR-associated EPSCs or IPSCs were measured using Stimfit (Guzman et al., 2014). To quantify the relationship between amplitude of SWRs and the corresponding magnitude of SWR-associated synaptic conductances, we calculated the maximal positive ripple amplitude in the band pass-filtered LFP and the peak of the associated EPSG or IPSG in each SWR event. To increase the reliability of this analysis, we only considered cells in which small SWR events (4 SD < RMS power < 7 SD) coexisted with large SWR events (RMS power ≥ 7 SD).

Derivative-based analysis of postsynaptic currents

To quantitatively examine the temporal structure and relationship between SWR-associated synaptic conductances and ripple oscillations *in vivo*, we used a first derivative-based detection method (Maier et al., 2011). The method exploits the characteristic kinetic property of synaptic currents, which is a fast rise followed by a slower decay. For derivative analysis, we first processed SWR-associated EPSCs or IPSCs with a 0.5–400-Hz band pass filter (zero-phase 2nd order Butterworth; using the filtfilt function of Matlab). Next, we calculated the first derivatives of the resulting traces, and marked their extrema in both upward and downward directions within a ±50 ms window around a SWR maximum. Finally, we detected EPSC onsets as derivative minima, and IPSC onsets as derivative maxima. To account for variability in the kinetic properties of synaptic currents, we analyzed different subsets of data representing the largest derivative peaks (Maier et al., 2011). The values of computed parameters (phase relations, IPSC-triggered LFP averages) remained consistent over a wide range of percentiles examined (1–25%).

To quantitatively describe the temporal relationship between fast LFP oscillations and synaptic dynamics on a “ripple cycle-by-cycle” basis, we first computed the Hilbert transform of the band pass-filtered LFP signal (100–250 Hz, FFT filter;

English et al., 2014). Next, each EPSC or IPSC onset time point was assigned a Hilbert phase value, φ_j. Finally, the mean phase angle was obtained for each cell, and averaged across cells. EPSC/IPSC-triggered averages of SWRs were computed by averaging ±50-ms stretches of LFP recording centered at the times of 10% largest derivative minima (EPSC onset) or derivative maxima (IPSC onset). Extracted LFP recordings were filtered in 0.5–400 Hz (wide band) or 100–250 Hz (ripple band) for illustrative purposes.

Simulations

To quantitatively assess voltage-clamp and event detection errors, CA1 pyramidal neurons recorded *in vivo* were traced using a digital reconstruction system (Neurolucida 9.0; Microbrightfield) and 60 x / 1.4 numerical aperture (NA) or 100 x / 1.4 NA oil-immersion objectives. Only cells in which the dendritic tree of the postsynaptic cell appeared largely intact were included in the analysis. To estimate voltage-clamp errors, we simulated EPSCs and IPSCs in realistic cable models of CA1 pyramidal neurons. After reconstruction of dendritic and axonal morphology, Neurolucida data were imported into Neuron 7.4 (Carnevale and Hines, 2006). The number of segments per section was set according to the d_lambda rule, so that the length of each segment was < 3.3% of length constant λ at 1000 Hz. The integration time step was set to 5 µs. Cable parameters were R_i = 150 Ω cm, R_m = 30,000 Ω cm^2, and C_m = 0.8 µF cm^{-2}. Synaptic conductances were simulated as double exponential functions, with rise time constant τ_{rise} (0.2 ms), decay time constant τ_{decay} (2.5 ms for excitatory synapses, 7.5 ms for inhibitory synapses), and reversal potential E_{syn} (0 mV for excitatory synapses, −65 mV for inhibitory synapses). Voltage-clamp conditions were established inserting a SEClamp point process at the center of the soma, assuming a series resistance of 10 MΩ.

For simulation of voltage-clamp errors (Jonas et al., 1993; Major et al., 1994; Williams and Mitchell, 2008; **Figure S3–S5**), synaptic peak conductance was assumed as 1 nS. Synapses were sequentially placed on all dendritic segments, and somatic EPSCs and IPSCs were simulated as the clamp current (i) of the SEClamp point process. For simulation of detection errors (**Figure S7**), peak conductance amplitudes were assumed to have a coefficient of variation of 0.6; mean peak conductance was 0.75 nS for excitatory and 1.5 nS for inhibitory events. Excitatory synapses were placed on dendrites < 300 µm from the center of the soma, approximately corresponding to

Schaffer collateral input. Inhibitory synapses were placed on dendrites < 150 μm from the center of the soma, approximately corresponding to inputs from basket or bistratified cells. Synaptic events were either simulated with random time points (25% of events), or with SWR temporal structure as a series of Gaussian functions with 8 ms spacing, with standard deviation of 1 ms, and amplitudes defined by a Gaussian envelope function, with standard deviation of 12 ms (75% of events). Gaussian white noise was added to the data, and simulated traces were filtered before analysis, similar to the experimental traces (400 Hz; 2nd order Butterworth characteristics). Events were classified as true positive if the time difference between detected and the nearest original event was in the range ±1 ms (after correction for the delay between onset and steepest point in the rising phase of a simulated EPSC or IPSC).

Statistics

Data are presented as means ± standard error of the mean (SEM). Differences were examined for statistical significance using a non-parametric Wilcoxon signed-rank test; differences were considered statistically significant if $P < 0.05$. Circular uniformity in phase relationship analysis was examined with a Rayleigh test (Zar, 2010). Throughout the paper, * indicates $P < 0.05$, ** $P < 0.01$, and *** $P < 0.001$.

Supplemental References

Bormann, J., Hamill, O.P., and Sakmann, B. (1987). Mechanism of anion permeation through channels gated by glycine and γ-aminobutyric acid in mouse cultured spinal neurones. J. Physiol. *385*, 243–286.

Carnevale, N.T., and Hines, M.L. (2006). The Neuron Book. (Cambridge, UK: Cambridge University Press).

Guzman, S.J., Schlögl, A., and Schmidt-Hieber, C. (2014). Stimfit: quantifying electrophysiological data with Python. Front. Neuroinform. *8*, 16.

Han, X., Chow, B.Y., Zhou, H., Klapoetke, N.C., Chuong, A., Rajimehr, R., Yang, A., Baratta, M.V., Winkle, J., Desimone, R., and Boyden, E.S. (2011). A high-light sensitivity optical neural silencer: development and application to optogenetic control of non-human primate cortex. Front. Syst. Neurosci. *5*, 18.

Hippenmeyer, S., Vrieseling, E., Sigrist, M., Portmann, T., Laengle, C., Ladle, D.R., and Arber, S. (2005). A developmental switch in the response of DRG neurons to ETS transcription factor signaling. PLoS Biol. *3*, e159.

Jonas, P., Major, G., and Sakmann, B. (1993). Quantal components of unitary EPSCs at the mossy fibre synapse on CA3 pyramidal cells of rat hippocampus. J. Physiol. *472*, 615–663.

Paxinos, G., and Franklin, K.B.J. (2001). The mouse brain in stereotaxic coordinates, 2nd edition. (San Diego, California: Academic Press).

Pernía-Andrade, A.J., Goswami, S.P., Stickler, Y., Fröbe, U., Schlögl, A., and Jonas, P. (2012). A deconvolution-based method with high sensitivity and temporal resolution for detection of spontaneous synaptic currents in vitro and in vivo. Biophys. J. *103*, 1429–1439.

Pernía-Andrade, A.J., and Jonas, P. (2014). Theta-gamma-modulated synaptic currents in hippocampal granule cells in vivo define a mechanism for network oscillations. Neuron *81*, 140–152.

Zar, J.H. (2010). Biostatistical analysis, 5th edition. (Upper Saddle River, New Jersey: Prentice Hall).
